# Integrated Enrichment Analysis of Variants and Pathways in Genome-Wide Association Studies Indicates Central Role for IL-2 Signaling Genes in Type 1 Diabetes, and Cytokine Signaling Genes in Crohn's Disease

**DOI:** 10.1371/journal.pgen.1003770

**Published:** 2013-10-03

**Authors:** Peter Carbonetto, Matthew Stephens

**Affiliations:** 1 Dept. of Human Genetics, University of Chicago, Chicago, Illinois, United States of America; 2 Dept. of Statistics, University of Chicago, Chicago, Illinois, United States of America; University of Oxford, United Kingdom

## Abstract

Pathway analyses of genome-wide association studies aggregate information over sets of related genes, such as genes in common pathways, to identify gene sets that are *enriched* for variants associated with disease. We develop a model-based approach to pathway analysis, and apply this approach to data from the Wellcome Trust Case Control Consortium (WTCCC) studies. Our method offers several benefits over existing approaches. First, our method not only interrogates pathways for enrichment of disease associations, but also estimates the level of enrichment, which yields a coherent way to promote variants in enriched pathways, enhancing discovery of genes underlying disease. Second, our approach allows for multiple enriched pathways, a feature that leads to novel findings in two diseases where the major histocompatibility complex (MHC) is a major determinant of disease susceptibility. Third, by modeling disease as the combined effect of multiple markers, our method automatically accounts for linkage disequilibrium among variants. Interrogation of pathways from eight pathway databases yields strong support for enriched pathways, indicating links between Crohn's disease (CD) and cytokine-driven networks that modulate immune responses; between rheumatoid arthritis (RA) and “Measles” pathway genes involved in immune responses triggered by measles infection; and between type 1 diabetes (T1D) and IL2-mediated signaling genes. Prioritizing variants in these enriched pathways yields many additional putative disease associations compared to analyses without enrichment. For CD and RA, 7 of 8 additional non-MHC associations are corroborated by other studies, providing validation for our approach. For T1D, prioritization of IL-2 signaling genes yields strong evidence for 7 additional non-MHC candidate disease loci, as well as suggestive evidence for several more. Of the 7 strongest associations, 4 are validated by other studies, and 3 (near IL-2 signaling genes *RAF1*, *MAPK14*, and *FYN*) constitute novel putative T1D loci for further study.

## Author Summary

Genome-wide association studies have helped locate gene variants that affect our susceptibility to diseases. The analysis of these studies is typically straightforward: test each genetic variant whether it is correlated with predisposition to disease. This approach often works well for identifying commonly occurring variants with moderate effects on disease risk. However, the effects of many variants are so small they fail to register statistically significant correlations. This is a concern because many diseases are modulated by many genetic factors with small effects on disease risk. An alternative is to examine groups of variants, such as variants sharing a common pathway, and assess whether these groups are “enriched” for correlations with disease. This can be a more effective approach to identifying genetic factors relevant to disease. However, it does not tell us which genes are associated with disease. To address this limitation, we describe an approach that integrates enrichment analysis with tests for disease-variant correlations within a single framework. We illustrate this approach in genome-wide studies of seven complex diseases. We show that our approach supports enriched pathways in several diseases, and uncovers disease-susceptibility genes in these pathways not identified in conventional analyses of the same data.

## Introduction

By systematically surveying the genome for variants correlated with disease phenotypes, genome-wide association studies (GWAS) have led to the discovery of genes and genetic loci underlying complex diseases [1]–[4]. Even though disease-correlated variants tend to have small effects on susceptibility to disease, followup investigations of the genetic loci implicated by GWAS have nonetheless advanced our understanding of many complex diseases. One strategy researchers have taken to translate initial genetic clues into biological models of disease etiology has been to identify common features of these genetic loci. For example, the discovery of disease-correlated variants in GWAS of Crohn's disease, a common form of inflammatory bowel disease, has helped draw links to genes that regulate autophagy and innate immune responses [5]–[10].

Recognizing that insights into disease can emerge by exploring the functional relationships among genes implicated in GWAS, researchers have attempted to assess these relationships in a systematic way by developing “pathway analysis” approaches to GWAS [11]–[28]. These methods are motivated by the theory that complex disease arises from the accumulation of genetic effects acting within common biological pathways [29]–[32]. The aim is to identify pathways that are *enriched* for disease—that is, groups of related genes that preferentially harbour disease-associated variants compared to arbitrary regions of the genome. Identifying enriched pathways is an important aim in itself, but pathway analysis can also improve power to uncover genetic factors relevant to disease; a major shortcoming of standard mapping approaches that test each marker one at a time for association with disease is that they lack power to map genetic factors of small effect [33]–[36]. The intuition is that identifying the accumulation of genetic effects acting in a common pathway is often easier than mapping the individual genes within the pathway that contribute to disease susceptibility.

Despite the considerable potential of pathway analysis approaches to GWAS, existing methods have an important limitation: they do not tell us which genes within an enriched pathway are most likely relevant to disease. Identifying enriched pathways is often useful, but many pathways contain genes with only loosely interrelated functions, so identifying the genes and variants within the pathway that are driving the enrichment is likely to yield additional insights into disease. This could be tackled in a two-stage process: first, identify the enriched pathways; second, gauge support for associated variants within the enriched pathways. In the second stage, significance thresholds for association could be relaxed relative to a genome-wide scan, reflecting the increased likelihood that variants near genes in the pathway are associated with disease. This is called *prioritizing* variants within the pathway [29], [37]–[45]. The question is how to implement this in a systematic way: to what extent can we relax significance thresholds while keeping the rate of false positives at an acceptable level?

To address this question, we develop a model-based approach for integrated analysis of pathways and genetic variants, in which we interpret enrichment as a parameter of the model. We begin with a large-scale multivariate regression that models disease risk as the combined effect of multiple markers. Unlike single-marker disease mapping, the multi-marker approach accounts for correlations between variants that arise due to linkage disequilibrium. Within this framework, we introduce an enrichment parameter that quantifies the increase in the probability that each variant in the pathway is associated with disease susceptibility. This model-based approach not only estimates the level of enrichment, but also adjusts the evidence for disease associations in light of predicted pathway enrichments—and, in so doing, tackles the problem of how to prioritize variants related to groups of genes or pathways.

Though we focus on incorporating pathways—and, more broadly, biologically related gene sets—into analysis of GWAS, our methods could be applied to other types of genome annotations, such as Gene Ontology categories [46], and DNA sequences where proteins are recruited to regulate gene transcription [47]–[51]. In this respect, our method is related to other model-based approaches that leverage prior knowledge about variants to estimate enrichment of association signals across functionally related regions or locate causal variants that affect disease risk [29], [52]–[62]. One distinguishing feature of our approach is that we have an efficient procedure to evaluate hypotheses about enrichment, which allows us to interrogate support for enrichment of thousands of candidate pathways in genome-wide data.

Another feature that distinguishes our analysis is that we use multiple pathway databases in an attempt to interrogate pathways as comprehensively as possible—the more pathways we consider, the greater chance we have of drawing new connections between pathways, genes within these pathways, and complex disease. We demonstrate how using our approach to comprehensively interrogate pathways results in increased evidence for enrichment, and is robust to inclusion of a large number of irrelevant pathways. In our case studies, we include ∼3100 candidate gene sets drawn from eight pathway databases available on the Web [44], [63], [64].

We illustrate our approach in a detailed analysis of genome-wide data from the Wellcome Trust Case-Control Consortium (WTCCC) studies of 7 complex diseases [65]. These studies provide an opportunity to gauge the added value of our approach because genetic associations based on these data have already been published [65], and pathway analyses of these data have found evidence for enriched pathways [11], [13], [16], [22], [23], [66]–[74]. Our methods highlight several pathways that have not been identified in previous pathway-based analyses, but which are known to be linked to these diseases. And, by prioritizing variants within the enriched pathways, our methods identify disease-susceptibility candidates that are not deemed significant in conventional analyses of the same data. These results demonstrate the potential for our methods to yield novel biological insights into complex disease.

### Overview of statistical analysis

Our approach builds on previous work that casts simultaneous analysis of genetic variants as a *variable selection problem*—the problem of deciding which variables (the genetic variants) to include in a multivariate regression of the phenotype. We begin with a method that assumes each variant is equally likely to be associated with the phenotype [75], [76], then we modify this assumption to allow for enrichment of associated variants in a pathway.

The data from the GWAS are the genotypes 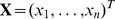 and phenotypes 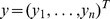 from *n* study participants. We assume the genetic markers are single nucleotide polymorphisms (SNPs), and the phenotype is disease status: patients with the disease (“cases”) are labeled 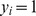, and disease-free individuals (“controls”) are labeled 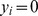. Entries of the 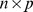 matrix 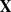 are observed minor allele counts 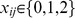, or expectations of these counts estimated using genotype imputation [77], [78], for each of the *n* samples and *p* SNPs.

We assume an additive model of disease risk, in which the log-odds for disease is a linear combination of the minor allele counts:
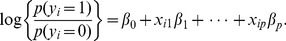
(1)Under this additive model, 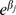 is the *odds ratio*, the multiplicative increase in odds of disease for each copy of the minor allele at locus *j*. We do not consider dominant or recessive effects on disease risk, but it would be straightforward to include them; see [79]. This method is also easily adapted to quantitative traits by replacing (1) with a linear regression for *y*.

Although the log-odds for disease is expressed in (1) as a linear combination of all SNPs, our framework is guided by the assumption that most SNPs have no effect on disease risk (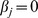). While there is some debate over this assumption [80], an advantage of this choice is that a SNP “included” in the multi-marker disease model—that is, a SNP *j* that has a non-zero coefficient, 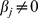
—indicates that the SNP is relevant to disease, or that it is in linkage disequilibrium with other, possibly untyped, variants that contribute to disease risk. Therefore, the main goal of the analysis is to identify regions of the genome that contain SNPs included in the disease model with high posterior probability, or identify SNPs within these regions that have a high “posterior inclusion probability,” 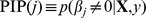. A high PIP is the analogue of a small *p*-value in a conventional single-marker analysis.

To obtain these posterior probabilities, we must first specify a prior for the coefficients 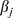. A standard assumption, and the assumption made in previous approaches [75], [76], is that all SNPs are equally likely to be associated with the phenotype *a priori*; that is, 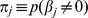 is the same for all SNPs.

To model enrichment of associations within a pathway, we modify this prior. Precisely, the prior inclusion probability for SNP *j* depends on whether or not it is assigned to the enriched pathway:
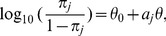
(2)where the pathway indicators 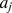 record which SNPs are assigned to the enriched pathway; 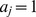 when SNP *j* is assigned to the enriched pathway, otherwise 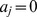. (In brief, a SNP is assigned to a pathway if it is near a gene in the pathway; see Methods.) We refer to 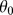 as the *genome-wide log-odds*, since it reflects the background proportion of SNPs that are included in the multi-marker disease model. (More precisely, it is the proportion corresponding to SNPs not assigned to the pathway, which is usually most SNPs.) We refer to 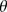 as the *log-fold enrichment* because it corresponds to the increase in probability, on the log-odds scale, that a SNP assigned to the pathway is included in the model. For example, 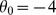 and 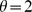 indicates that 1 out of every 10,000 SNPs outside the pathway is included in the multi-marker model, but for SNPs assigned to the pathway, 1 out of every 100 is included. If 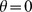, this reduces to the standard prior assumption made by previous methods. We expect 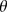 to be 0, or close to 0, for most pathways.

We assess enrichment by framing each hypothesis test for enrichment as a model comparison problem. To weigh the evidence for the hypothesis that candidate pathway with indicators 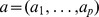 is enriched for disease associations, we evaluate a *Bayes factor* [81], [82]:
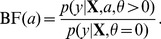
(3)This Bayes factor (BF) is the ratio of likelihoods under two models, the model in which the candidate pathway is enriched for SNPs included in the multi-marker model (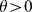), and the null model with no enrichment (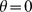). A larger BF implies stronger evidence for enrichment. We compute each BF by averaging, or *integrating*, over the unknown parameters, and over multi-marker models with different combinations of SNPs, employing appropriate prior distributions for 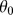, 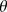, and the coefficients 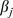 (see Methods).

Note that the Bayes factor (3) does not allow for a negative 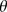
—that is, pathways that are *underrepresented* for associations with the phenotype. While it could be useful to investigate negative log-enrichments in other settings, in most GWAS of complex disease where there are generally few significant associations to begin with, reduced rates of disease associations in pathways would be difficult to find, and would be unlikely to have a useful interpretation.

We use the same approach to test for joint enrichment of multiple candidate pathways. We compute 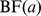 as before (eq. 3), except that we set 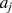 to 1 whenever SNP *j* is assigned to at least one of the enriched pathways. In this case, 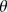 represents the increased level of associations (on the log-odds scale) among SNPs assigned to one or more of the pathways. This is equivalent to assuming that all enriched pathways have the same level of enrichment, which greatly simplifies the analysis. We allow for different enrichment levels only when accounting for enrichment of the MHC in RA and T1D. In that case, we have good reason to treat the MHC differently, given the predominant contribution of MHC alleles to RA and T1D risk [83], [84].

To assess evidence for association of individual variants with the phenotype, we compute 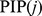 for each variant *j*. These posterior probabilities depend on which pathways are enriched, and on the log-fold enrichment 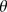, because these factors affect the prior probabilities 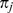, which in turn affect the posterior probabilities 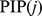, following Bayes' rule. (In practice, we account for uncertainty in 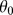 and 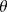 when calculating the posterior probabilities by averaging over 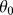 and 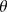; see Methods.) Since enrichment leads to higher prior inclusion probabilities for SNPs in the enriched pathway, an association that is not identified by a conventional genome-wide analysis may become a strong candidate in light of its presence in an enriched pathway. Because we estimate 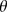 from the data, the extent to which we prioritize variants is determined by the data. In this way, our framework integrates the problem of identifying enriched pathways with the problem of prioritizing variants near genes in enriched pathways.

## Results

We illustrate our methods in a detailed analysis of genome-wide marker data from case-control studies of seven common diseases: bipolar disorder (BD), coronary artery disease (CAD), Crohn's disease (CD), hypertension (HT), rheumatoid arthritis (RA), type 1 diabetes (T1D) and type 2 diabetes (T2D) [65]. After steps to ensure data quality (see Methods), the data for each disease consist of ∼440,000 SNPs genotyped for 1748–1963 cases and 2938 controls (Table S1). Many of the genetic associations based on these data [65] have been replicated in follow-up studies [85]–[89]. We compare our results to previously reported associations, and to existing pathway analyses of these data [11], [13], [16], [22], [23], [66]–[74].

We analyze the data in three stages. First, we compute a BF for each candidate pathway to assess whether it is enriched for disease associations, and we rank the pathways according to their BFs. (Throughout, we use “pathway” to refer to a collection of functionally related genes.) Second, we investigate whether prioritizing variants within the enriched pathways can help locate disease associations beyond those identified in analyses that ignore information about pathways. Finally, for diseases with evidence of pathway enrichment, we re-examine the data for models in which two or more pathways are enriched, and investigate whether prioritizing combinations of enriched pathways yields further disease associations.

### Selection of candidate pathways

We assemble a comprehensive list of candidate pathways to test for enrichment, drawing from a variety of publicly accessible collections (see Methods). We do not filter pathway candidates based on their potential relevance to disease. In total, we interrogate 3158 candidate pathways for each disease, plus the MHC and ×MHC gene sets described below. Most candidate pathways were curated by domain experts, and others are based on experimental evidence in non-human organisms and inferred via gene homology. For full details of pathway databases used, and steps taken to compile gene sets from pathway data, refer to Methods, Supplementary Materials, and links to source code implementing our analyses.

### The major histocompatibility complex

In two of the seven diseases, RA and T1D, multiple disease associations map to the major histocompatibility complex (MHC) region on chromosome 6. Consequently, pathway analyses for RA and T1D tend to highlight pathways that involve MHC genes. When we apply our method to these diseases, the top pathways for T1D and RA are “Allograft rejection” and “Asthma,” respectively. Both gene sets include multiple MHC genes, and exhibit strong evidence for enrichment (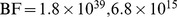). Other pathways with the strongest enrichment signals also contain MHC genes.

Most of the support for enrichment of these pathways is likely driven by disease associations that map to the MHC. To check this, we create a “pathway” containing all genes within the MHC [90], and test this gene set for enrichment. The MHC gene set shows more support for enrichment than any other pathway by several orders of magnitude (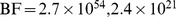 in T1D and RA, respectively), and it is accompanied by a high enrichment estimate (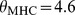 and 3.7). Performing a similar enrichment analysis for all genes within the “extended” MHC (xMHC) [91] yields smaller BFs (Figure S1), suggesting that the genetic contribution to RA and T1D risk lies mostly within the class I, II and III subregions of the MHC.

Our finding that the MHC is enriched for associations with T1D and RA is unsurprising considering the MHC is estimated to account for over half the genetic contribution to T1D risk, and at least a third for RA [83], [84], [92]. (By contrast, the genetic contribution of the MHC is estimated to be ∼10% for CD [83], and the BFs for the MHC and ×MHC in CD are 8 and 4, respectively.) In light of these findings, a reasonable question to ask is whether pathways show enrichment for disease associations beyond enrichment of the MHC. A strength of our model-based approach is that it can address this question by computing a BF for enrichment of each candidate pathway, conditioned on the estimated enrichment of the MHC. Thus, in our subsequent analysis of RA and T1D, we account for enrichment of disease associations within the MHC in this way. As far as we are aware, no other pathway-based analyses of these data incorporate enrichment of the MHC, which may explain why previous studies have highlighted mostly MHC-related pathways and gene categories.

### Bayes factors for enrichment

**Figure 1:**
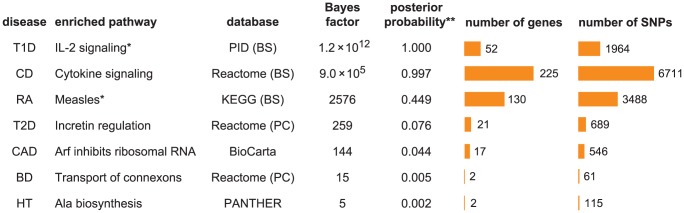
Diseases show a wide range of support for enrichment of disease associations in pathways. Each row shows the pathway with the largest BF for enrichment of disease associations among 3158 candidate gene sets. Columns left to right: (1) disease; (2) enriched pathway; (3) pathway database, and repository where pathway is retrieved if different from database; (4) BF for hypothesis that disease associations are enriched among SNPs assigned to pathway; (5) posterior probability of enrichment hypothesis; (6) number of genes assigned to pathway; (7) number of SNPs near these genes. Abbreviations used in figure: PID = NCI Nature Pathway Interaction Database [163], BS = NCBI BioSystems [164], PC = Pathway Commons [165]. Databases and database identifiers for pathways listed here: “Transport of connexons to the plasma membrane” (Reactome 11050, PC); “Tumor suppressor Arf inhibits ribosomal biogenesis” (BioCarta); “Cytokine signaling in immune system” (Reactome 75790, BS 366171); “Alanine biosynthesis” (PANTHER P02724); “Measles” (KEGG hsa05162, BS 213306); “IL2-mediated signaling events” (PID il2_1pathway, BS 137976); “Incretin synthesis, secretion, and inactivation” (Reactome 23974, PC). *Null and enrichment hypotheses for RA and T1D include enrichment of disease associations in MHC, in which SNPs within MHC are enriched at a different level than non-MHC SNPs in pathway; 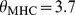 and 4.6 for RA and T1D, respectively. Number of genes/SNPs for RA and T1D count only non-MHC genes assigned to pathway. **Illustrative posterior probability assuming a “conservative” prior (see text).

To give an initial impression of our enrichment results, we show the pathway with the largest BF for each disease in Figure 1. The seven diseases exhibit a wide range of support for the strongest enrichment signal. For example, the top pathway for T1D, IL-2 signaling, has a BF of 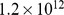, whereas the largest BF for HT is only 5.

To address whether these top pathways constitute “significant” evidence for enrichment, the BF for enrichment must be weighed against the prior probability of the pathway being enriched to obtain a posterior probability of enrichment (see “Interpretation of Bayes factors” in Methods). While specification of a prior probability of enrichment is subjective, this subjectivity is unavoidable; similar issues arise when specifying significance thresholds for *p*-values, though these issues are usually hidden (0.05 is a common threshold, but it is subjective and not universally appropriate [93]). If we apply a “conservative” value of 1/3158 to the prior probability for all candidate pathways, so that one pathway is expected to be enriched among the 3158 candidates, then CD and T1D show compelling evidence for enrichment (posterior probability>0.99), and RA shows suggestive evidence (posterior probability = 0.45). Considering the plausible connection between Measles pathway genes and RA (discussed below), we view this as a compelling enrichment as well. The top pathway for T2D, Incretin regulation, shows only modest evidence for enrichment if we apply the conservative prior, but it might be considered “significant” if we adopt a less conservative prior to account for the known connection of this pathway to insulin resistance and diabetes. Based on these results, we do not investigate BD, CAD and HT further, and focus on the four diseases showing strongest evidence for enrichment, CD, RA, T1D and T2D.

**Figure 2:**
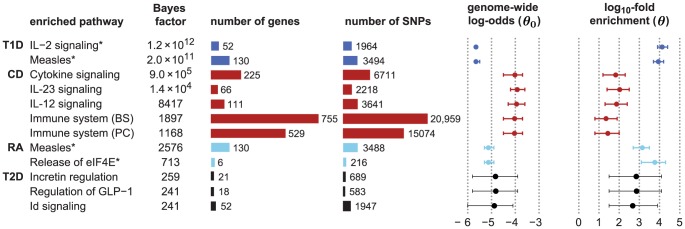
Top-ranked candidate pathways for enrichment of disease associations in CD, RA, T1D and T2D. Refer to Figure 1 for legend, abbreviations, and meaning of asterisk (*). Two right-most columns show posterior mean and 95% credible interval of genome-wide log-odds (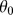) and log-fold enrichment (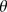) given that pathway is enriched (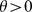). Note that enrichment level is defined on log-scale (eq. 2), so 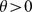 indicates enrichment. Credible interval is smallest interval about mean that contains parameter with 95% posterior probability, calculated to nearest 0.1 using a numerical approximation. Database identifiers for pathways not previously mentioned: “IL23-mediated signaling events” (PID il23pathway, PC); “IL12-mediated signaling events” (PID il12_2pathway, PC); “Immune system” (Reactome 6900, BS 106386); “Release of eIF4E” (Reactome 6836, PC); “Synthesis, secretion, and inactivation of glucagon-like peptide-1” (Reactome 24019, PC); “Id signaling pathway” (WikiPathways WP53 [166], BS 198871). See Figure S1 for more gene set enrichment results.

Figure 2 shows an expanded list of pathways with the strongest support for enrichment in CD, RA, T1D and T2D, together with estimated enrichment levels (see Figure S1 for a longer list). Beyond these top results, the vast majority of candidate pathways show little or no evidence for enrichment (Figure S2), demonstrating that the method is robust to inclusion of many pathways that are most likely irrelevant to the disease.

Before discussing the biological relevance of these pathways, we point out three general features of Figure 2. First, some of the estimated enrichments are extremely large; for example, IL-2 signaling genes show more than a 10,000-fold enrichment of T1D risk factors. In contrast, the top pathway for CD, “Cytokine signaling in immune system,” has roughly a 100-fold enrichment. (Enrichment of this pathway nonetheless yields a large BF, partly because it implicates over 6700 SNPs; the BFs depend not only on the level of enrichment, but also on the number of SNPs assigned to the pathway.) Second, some of the top pathways overlap or are subsets of one another. For example, “Cytokine signaling in immune system” is a subset of “Immune system.” Also, the Immune system pathway from NCBI BioSystems (BS) overlaps with the Pathway Commons (PC) version of the same pathway (510 genes are common to both gene sets). This raises the question whether enrichment of just one pathway would suffice to explain the genome-wide association signal; we use our methods to investigate this question below. Third, 5 different pathway databases are represented in Figure 1, and all 8 pathway databases included in our analysis appear among the top pathways (Figure 2), illustrating the benefits of interrogating pathways from multiple sources.

### Biological relevance of enriched pathways

The top-ranked pathway for CD (“Cytokine signaling”) is a collection of cytokine-driven networks that exhibit a complex relationship to autoimmunity—they promote inflammatory and immune responses, while also playing an important role in suppressing immunity [94]. Cytokine signaling implicates a broad class of 225 genes, suggesting that a collection of related gene networks explains the pattern of genetic associations better than any one signal transduction pathway. Enrichment of cytokine signaling is consistent with the accumulating evidence that points to cytokines, and the signaling cascades initiated by these cytokines, in a range of autoimmune disorders, including inflammatory bowel disease [9], [95], [96].

Previous findings from GWAS have linked autophagy genes *ATG16L1* and *IRGM* to CD [65], [97], [98]. Our pathway analysis does not provide additional support for autophagy in CD because pathways reflecting current models of autophagy [9], [99] have not yet been incorporated, as far as we are aware, into any of the publicly available pathway databases.

Once we account for enrichment of the MHC, the top pathway for T1D is IL-2 signaling. Cytokine IL-2 and its interacting partners are indispensable to activation, development and maintenance of T regulatory cells, and disruption of IL2-mediated pathways promotes progression of autoimmune disorders [100]–[102]. T1D treatments targeting the IL-2 signaling pathway are currently undergoing clinical trials [103]. Additionally, studies in non-obese diabetic (NOD) mice suggest that defects in IL-2 signaling induce susceptibility to T1D [102], [104], [105]. Our findings support this hypothesis.

The top pathway for RA, the KEGG “Measles” pathway, contains genes involved in immune response cascades triggered by infection of measles virus, including the cellular receptors expressed for measles virus such as *SLAM* and *CD46* [106]–[108]. (This result is again conditioned on enrichment of the MHC.) While studies have associated the measles virus with RA [109], other viral and bacterial infections have also been linked to incidence of RA [110], [111], and enrichment of the Measles pathway could reflect a larger class of genes involved in regulation of immune function during infection, rather than the measles virus specifically. The large BF for this pathway in both RA and T1D supports previous indications of a shared genetic basis [96], [112], and is consistent with observations that RA and T1D, along with other autoimmune diseases, recur in the same families [113].

All CD, RA and T1D pathways in Figure 2 implicate key actors in responses to pro-inflammatory stimuli and in regulation of innate and adaptive immunity. These include members of the NF-kB/Rel family, T-cell receptors (TCRs), members of the protein tyrosine phosphatase family (PTPs), mitogen-activated protein (MAP) kinases such as c-Jun NH2-terminal kinases (JNKs), and chemokine receptors (CXCRs) [114]–[117].

### Comparison with previous pathway analyses

None of the top-ranked pathways for CD, RA and T1D in our analysis have been identified in previous pathway-based analyses of these diseases [11], [13], . An important difference between our methods and previous pathway analyses of RA and T1D is that we incorporate enrichment of the MHC into models of enrichment. A previous analysis of RA [67] highlighted pathways “Bystander B cell activation” (BioCarta, their *p*-value = 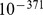) and “Type 1 diabetes mellitus” (KEGG, *p*-value = 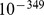). However, both these pathways contain MHC genes, and our results suggest that enrichment of the MHC offers a better explanation of the association signal; in our analysis, support for enrichment of these pathways is several orders of magnitude less than support for enrichment of the MHC (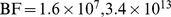 versus 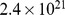), and the support vanishes once we account for enrichment of the MHC (BF = 0.69, 0.57). Similarly, previous analyses of T1D [16], [121] have highlighted the same “Type 1 diabetes mellitus” pathway, but again support for enrichment is driven mostly by the association signal in the MHC, as our methods yield only modest support for this pathway after accounting for MHC enrichment (BF = 43).

It is also notable that the top-ranked pathways for RA and T1D, Measles and IL-2 signaling, show strong support in our analysis *only after accounting for enrichment of disease associations within the MHC*; the BFs without MHC enrichment are 104 and 11, whereas the BFs are 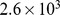 and 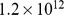 after conditioning on enrichment of the MHC. This may explain why these pathways have not been identified in previous pathway analyses of these diseases. These results illustrate the benefits of estimating enrichment conditioned on the MHC and, more generally, quantifying support for models with multiple enriched pathways.

Another aspect that differs between our results and previous studies is that we interrogate a more comprehensive set of pathway databases. This may explain in part why the BF for the top pathway in CD, “Cytokine signaling in immune system” from Reactome, eclipses the BFs corresponding to previously reported pathways. For example, Wang *et al* [73] interrogated BioCarta, KEGG and Gene Ontology [46] (and not Reactome) gene sets for enrichment of CD associations, and reported the smallest *p*-value for BioCarta pathway “IL12 and Stat4 dependent signaling in Th1 development” (*p*-value = 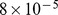, FDR = 0.045). This pathway showed little evidence for enrichment in our analysis (BF = 20) compared to cytokine signaling (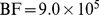). (Below, when we combine this pathway with cytokine signaling genes, we obtain stronger evidence for enrichment in CD; the BF is 81% the size of the largest BF for 2 enriched pathways.)

### Associations informed by enriched pathways

An important feature of our model-based approach is that pathway enrichments can help to map additional disease associations by prioritizing variants within enriched pathways. This is particularly useful for broad groups of enriched genes such as “Cytokine signaling in immune system,” which contains 225 genes, as only a small portion of these genes may actually harbour genetic variants that affect CD risk. Prioritization occurs automatically within our statistical framework; the enrichment parameter affects the prior probability of association for SNPs in the pathway, which in turn increases the posterior probability of association for these SNPs.

We therefore examine how re-interrogation of SNPs for association in light of inferred enrichments in CD, RA, T1D and T2D can reveal additional associations across the genome. We assess evidence for associations across genomic regions, rather than individual SNPs. The rationale is that genome-wide mapping using a multi-marker disease model sometimes spreads the association signal across nearby SNPs when they are correlated with one another, thereby diluting the signal at any given SNP [76]. We divide the genome into overlapping segments of 50 SNPs, with an overlap of 25 SNPs between neighbouring segments. For each segment, we compute 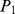, the posterior probability that at least one SNP in the segment is included in the multi-marker disease model. (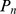 denotes the posterior probability that at least *n* SNPs are included.) We use segments with an equal number of SNPs so that, under the null hypothesis of no enrichment, the prior probability that at least one SNP is included is the same for each segment. A segment spans, on average, 307 kb (98% of the segments are between 100 kb and 1 Mb long), so calculating 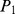 for these segments provides only a low-resolution map of genetic risk factors for disease. Still, this resolution suffices for our study.

**Figure 3:**
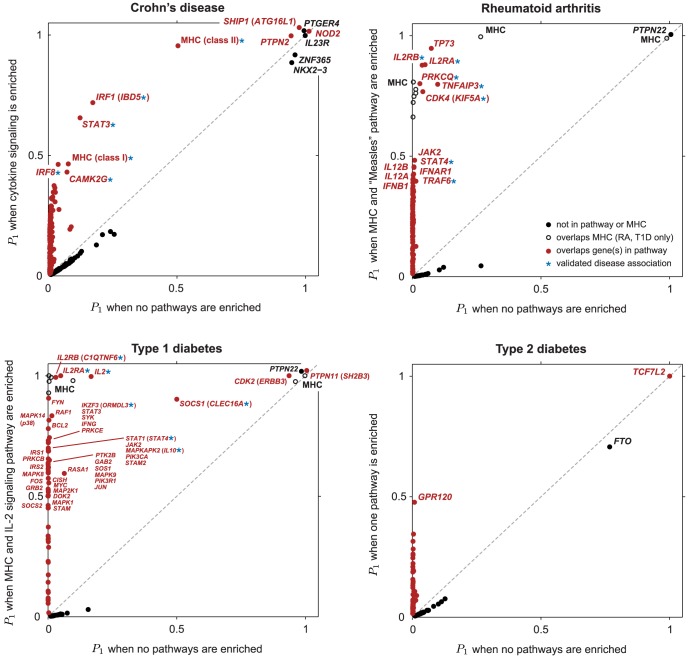
Scatterplots showing 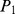, posterior probability that region contains disease risk variants, given different enrichment hypotheses. Each point corresponds to a small region of the genome containing 50 SNPs. Posterior probabilities on vertical axis for CD, RA and T1D are conditioned on enrichment of pathway with largest BF (Figure 1). For T2D, since no single pathway stands out in ranking (Figures 2 and S1), 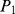 along vertical axis is obtained by averaging over top 5 pathways (see Methods). Points highlighted in red correspond to segments overlapping SNPs assigned to the enriched pathway (for T2D, at least 1 out of 5 top pathways). In RA and T1D, 50-SNP segments overlapping the MHC are drawn as open circles (SNPs in these segments are not assigned to the pathway). Overlapping segments sharing the same association signal are not shown. Some segments are labeled by gene(s) in pathway and/or most credible gene of interest based on prior studies (most credible gene is shown in parentheses if different from pathway gene). Asterisk (*) indicates an appreciable increase in the probability of a disease association, and this association is validated by other GWAS for same disease (see Table 1).

Table 1 summarizes the regions of the genome showing strongest evidence for association after pathway prioritization, and Figure 3 compares support for disease associations under the null hypothesis of no enrichment with support under the model in which the pathway with the largest BF is enriched.

**Table 1:** Regions of the genome exhibiting moderate to strong evidence for CD, RA and T1D risk factors.

				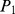		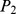		trend	Candidate gene(s)					MAF		corroborating
		chr.	region (Mb)	null	alt.	null	alt.	*p*-value [65]		SNP	PIP	LOR	(95% CI)	ctrls	cases	references
	CD	1p31	67.30–67.48	1.00	1.00	0.05	0.03	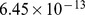	*IL23R*	rs11805303	1.00	0.25	*(0.18–0.33)*	*0.318*	*0.391*	**
	CD	2q37	233.92–234.27	1.00	1.00	0.01	0.05	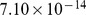	*ATG16L1* (***SHIP1***)	rs10210302	1.00	−0.27	(0.20–0.35)	0.481	0.402	**
	CD	5p13	40.32–40.66	1.00	1.00	0.46	0.39	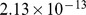	*PTGER4*	rs17234657	1.00	0.30	*(0.20–0.39)*	*0.124*	*0.181*	**
*	CD	5q23	129.54–132.04	0.28	**0.81**	0.04	0.40	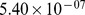	*IBD5* (***IRF1+3***)	rs274552	0.37	−0.14	(0.03–0.26)	0.166	0.128	[6], [172], [173]
*	CD	6	25.52–33.76	0.66	**1.00**	0.22	**0.95**	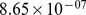	**MHC**	rs9469220	0.94	−0.16	(0.08–0.23)	0.519	0.465	[6], [83], [174]
	CD	10q21	64.0–64.43	0.96	0.92	0.04	0.02	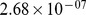	*ZNF365*	rs10995271	0.92	0.20	*(0.13–0.28)*	*0.386*	*0.440*	**
	CD	10q24	101.26–101.32	0.95	0.89	0.00	0.00	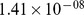	*NKX2-3*	rs7095491	0.88	0.19	*(0.11–0.26)*	*0.470*	*0.527*	**
	CD	16q12	49.0–49.4	1.00	1.00	0.16	**0.62**	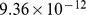	***NOD2***	rs17221417	1.00	0.24	(0.16–0.31)	0.287	0.356	**
*	CD	17q21	37.5–38.3	0.13	**0.67**	0.01	0.19	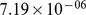	***STAT3***	rs744166	0.51	−0.14	(0.07–0.22)	0.439	0.392	[6], [7], [132]
	CD	18p11	12.76–12.91	0.94	1.00	0.00	0.11	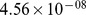	***PTPN2***	rs2542151	1.00	0.23	(0.13–0.31)	0.163	0.209	**
*	RA	1p36	3.5–3.7	0.07	**0.94**	0.00	0.16	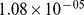	***TP73***	rs12027041	0.93	0.16	(0.08–0.23)	0.414	0.459	–
	RA	1p13	113.53–114.36	1.00	1.00	0.01	0.00	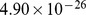	*PTPN22*	rs6679677	1.00	0.49	*(0.39–0.60)*	*0.096*	*0.169*	**
	RA	6	25.52–33.76	1.00	1.00	1.00	1.00	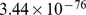	MHC	rs9268560	1.00	−0.31	(0.24–0.39)	0.483	0.306	**
*	RA	6q23	138.00–138.47	0.10	**0.80**	0.00	0.26	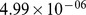	***TNFAIP3***	rs11970411	0.71	0.21	(0.08–0.31)	0.080	0.107	[88], [131], [175], [176]
*	RA	10p15	6.07–6.26	0.05	**0.87**	0.00	0.29	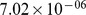	***IL2RA***	rs2104286	0.82	−0.16	(0.08–0.24)	0.286	0.244	[131], [176], [177]
*	RA	10p15	6.36–6.49	0.03	**0.76**	0.00	0.10	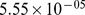	***PRKCQ***	rs1570527	0.73	−0.17	(0.08–0.26)	0.190	0.157	[85], [131], [176], [178]
*	RA	12q13	55.77–56.82	0.05	**0.77**	0.00	0.09	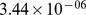	*KIF5A* **(** ***CDK4*** **)**	rs10876991	0.74	−0.15	(0.07–0.22)	0.347	0.307	[85], [131], [176], [178]
*	RA	22q13	35.57–35.90	0.05	**0.87**	0.00	0.14	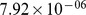	***IL2RB***	rs743777	0.85	0.16	(0.07–0.23)	0.294	0.336	[85], [131], [176]
	T1D	1p13	113.53–114.36	1.00	1.00	0.03	0.01	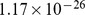	*PTPN22*	rs6679677	1.00	0.52	*(0.40–0.61)*	*0.096*	*0.170*	**
*	T1D	3p25	12.44–12.85	0.02	**0.85**	0.00	**0.57**	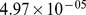	***RAF1***	rs299651	0.12	−0.09	(0.01–0.16)	0.512	0.475	–
*	T1D	4q27	123.3–123.93	0.17	**1.00**	0.00	0.27	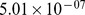	***IL2***	rs17388568	1.00	0.19	(0.10–0.27)	0.260	0.307	[130], [179]
	T1D	6	25.52–33.76	1.00	1.00	1.00	1.00	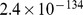	MHC	rs9273363	1.00	0.66	(0.59–0.73)	0.305	0.709	**
*	T1D	6p21	35.86–36.38	0.00	**0.82**	0.00	0.41	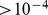	***p38***	rs2237093	0.58	−0.18	(0.07–0.30)	0.098	0.077	–
*	T1D	6q21	112.02–112.47	0.00	**0.95**	0.00	**0.77**	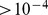	***FYN***	rs12910	0.66	−0.13	(0.06–0.21)	0.487	0.454	–
*	T1D	10p15	6.07–6.26	0.05	**1.00**	0.00	**0.70**	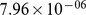	***IL2RA***	rs2104286	1.00	−0.20	(0.12–0.29)	0.286	0.245	[84], [130], [180]–[182]
	T1D	12q13	54.63–54.91	0.94	1.00	0.00	0.25	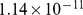	*ERBB3* (***CDK2***)	rs1873914	1.00	0.23	(0.15–0.30)	0.414	0.471	**
	T1D	12q24	109.8–111.74	1.00	1.00	0.01	**0.64**	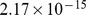	*SH2B3* **(** ***PTPN11*** **)**	rs17696736	1.00	0.32	(0.24–0.39)	0.424	0.505	**
*	T1D	16p13	10.9–11.41	0.51	**0.95**	0.01	**0.76**	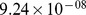	*CLEC16A* **(** ***SOCS1*** **)**	rs149310	0.61	0.14	(0.05–0.22)	0.248	0.284	[89], [130], [183]
*	T1D	22q13	35.68–36.0	0.03	**0.99**	0.00	**0.75**	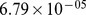	*C1QTNF6* **(** ***IL2RB*** **)**	rs3218253	0.97	0.18	(0.09–0.26)	0.251	0.286	[130], [184]

For all CD and RA loci in this table, there is at least a 0.5 probability that one or more SNPs in the region are included in the multi-marker disease model (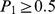); for all T1D loci, 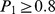. Support for disease associations is conditioned on enrichment of pathways in Figure 1. Rows marked with * are selected only after accounting for pathway enrichment, or show substantial increase in support due to feedback from enrichment. Right-most column cites published GWAS findings that corroborate majority of * rows. In this column, ** indicates that validation is not required as disease association is already strongly supported without pathways; these rows recapitulate the strongest associations reported in the original study [65] (see Supplementary Materials). Genes in enriched pathways are written in bold. Table columns from left to right are: (1) disease; (2) chromosomal locus; (3) region most likely containing the risk-conferring variant(s), in Megabases (Mb); (4) posterior probability that one or more SNPs in region are included in model under null, and (5) under enrichment hypothesis; (6) posterior probability that two or more SNPs are included under null, and (7) under enrichment hypothesis; (8) smallest trend *p*-value in region from original analysis [65], when available (some of these *p*-values are derived from imputed SNPs, and are not available in our data); (9) established genes in disease pathogenesis, or most credible genes of interest based on prior studies, corresponding to locus (when the most credible gene differs from gene assigned to pathway, pathway gene is shown in parentheses); (10) refSNP identifier of SNP in critical region with largest PIP (this SNP is likely in linkage disequilibrium with the causal variant rather than being causal itself, and may not match SNP reported in [65] with smallest *p*-value); (11) the PIP of this SNP; (12) posterior mean of log-odds ratio 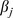 (additive effect of minor allele count on log-odds of disease) given SNP that is included in multi-marker disease model; (13) 95% credible interval of effect size, 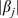; (14) frequency of minor allele for SNP in controls, and (15) in cases. Bold numbers in 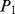 and 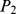 columns highlight appreciable increase in support for disease associations within region after feedback from enriched pathway. Credible interval is smallest interval about posterior mean that contains 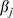 with 95% posterior probability. The “critical region” at each locus is estimated by inspecting single-SNP BFs [79], and bounding the region by areas of high recombination rate, inferred using data from Phase I, release 16a of the HapMap study [185], and visualized in UCSC Genome Browser [186]. Note that 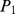 statistic for critical region may be slightly different than 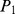 for overlapping segment shown in Figure 3 due to different numbers of SNPs in segments and critical regions. All SNP information and genomic positions are based on Human Genome Assembly 17 (NCBI build 35).

Overall, prioritization of SNPs in enriched pathways increases support for disease risk factors in many regions, often substantially; these regions correspond to points above the diagonal in the scatterplots (Figure 3). In CD and RA, 8 disease susceptibility loci with 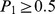, not including segments overlapping the MHC, are revealed only after prioritizing SNPs in enriched pathways. In T1D, prioritization of SNPs in the IL-2 pathway yields a total of 37 associated regions outside the MHC with 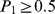. This dramatic result reflects the high estimated enrichment for IL-2 signaling genes. The majority of the additional disease regions with the strongest support, including many of the loci with weaker association signals, are validated by other studies; in CD and RA, 7 of the 8 additional disease susceptibility loci with 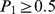 are corroborated by other GWAS and large-scale meta-analyses, and in T1D, 4 of the 7 additional disease regions with 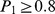 are similarly corroborated (see Table 1 for references).

Prioritization yields many new candidate disease susceptibility loci not previously implicated by GWAS. These loci will require followup studies to be validated. Three unconfirmed T1D susceptibility loci with strong support (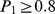) are regions containing IL-2 signaling genes *RAF1*, *MAPK14* and *FYN*: gene *RAF1* is a critical target of insulin in primary *β*-cells, and variants of this gene may modulate loss of *β*-cell mass in forms of diabetes [122]; *MAPK14* (*p38*) encodes at least 4 distinct isoforms, and deficiencies in one isoform have been shown in mice knockout studies to improve glucose tolerance and protect against insulin resistance, pointing to a role in development of T1D [123]; *FYN* interacts with *PTPN22* to regulate T cell receptor signaling, and *PTPN22* alleles are strongly associated with predisposition to T1D [124]. The one novel candidate region for RA contains Measles pathway gene *TP73*, whose homolog, *TP53*, is suspected to impair regulation of inflammation in RA patients [125], [126]. Finally, conditioning on enrichment of top pathways in T2D yields a single novel candidate region at 7q32 with moderate probability of containing a disease association (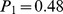). This region contains *GPR120* (also *OSFAR1*), a gene assigned to both the Incretin regulation and GLP-1 pathways. It was recently shown that *GPR120*-deficient mice develop obesity and reduced insulin signaling, and *GPR120* expression is significantly higher in obese humans [127], so the effect of this gene may be similar to the reported T2D association with *FTO*, in which variants near *FTO* increase T2D risk through an effect on body weight [128], [129].

In addition to these associated regions, several promoted regions also lie within the MHC. (In the scatterplots for RA and T1D, these regions correspond to open circles above the diagonal.) Given the complexity of this region, which contains a high density of genes, and long-range correlations between SNPs, disentangling the association signal in the MHC will likely require higher density SNP data, and lies beyond the capacities of our current implementation (see Discussion).

**Figure 4:**
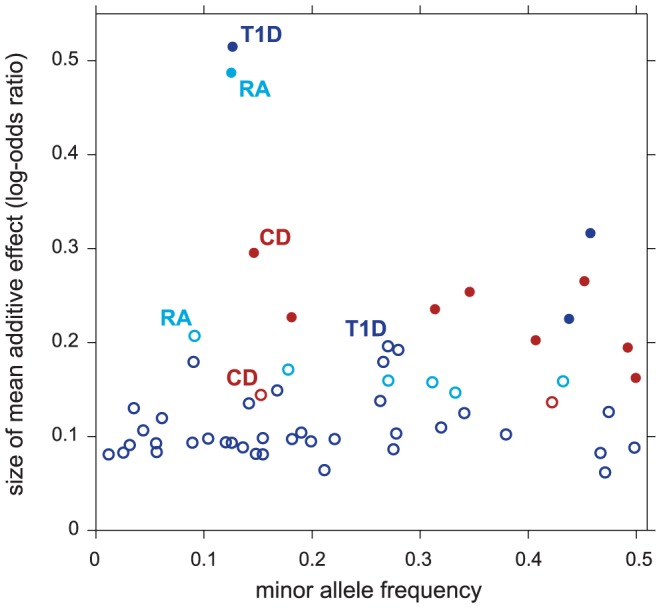
Variants in non-MHC disease regions revealed by enriched pathways have smaller effects on disease risk. Each point in scatterplot corresponds to a 50-SNP segment outside the MHC for which 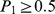. Filled circles correspond to selected regions containing disease risk factors without feedback from enriched pathways (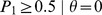); open circles correspond to selected regions conditioned on enrichment (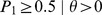 and 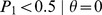). For each segment, minor allele frequency and posterior mean additive effect of minor allele count on log-odds of disease (“log-odds ratio”) are taken from SNP in segment with highest probability of being included in multi-marker model.

Figure 4 compares the effect sizes of variants in regions selected only after accounting for pathway enrichment to the effect sizes from regions identified without the benefit of feedback from pathway enrichment. As expected, pathway prioritization uncovers many disease-associated variants with smaller effects than we would otherwise be able to map reliably. This could explain, at least in part, why many of the putative T1D associations uncovered in our analysis are not yet confirmed; the largest meta-analysis of T1D to date, with a combined sample of size ∼16,000 [130], still has limited power to detect associations within this range of effect sizes and minor allele frequencies. (In contrast, much larger meta-analyses exist for CD and RA, with 30,000 and 47,000 samples, respectively [131], [132].)

Finally, Figure 3 offers the opportunity to remark on four other features of our results. First, the strongest association signals without feedback from pathways stay strong whether or not they are related to the enriched pathway—these associations correspond to the points in the top-right corner of each scatterplot. (Note that the segments in the top-right corner recapitulate the strongest associations reported for CD, RA, T1D and T2D in the original study [65]. See Supplementary Materials for a detailed comparison to single-marker *p*-values in all 7 diseases.) Second, many segments show slightly decreased support for association under the enrichment hypothesis (points below the diagonal in the scatterplots). This occurs because the estimated prior inclusion probability for SNPs outside the pathway is reduced to reflect the fact that pathway enrichment helps to explain an appreciable portion of the genome-wide association signal. Third, although not evident from the figure due to over-plotting, most segments show little or no evidence for associations under either hypothesis; in each scatterplot, 98–99.7% of segments lie near the bottom-left corner. Fourth, associations with strong support under the null are not necessary for establishing evidence for enriched pathways; none of the RA associations in the top-right corner of the scatterplot contribute to evidence for enrichment of the Measles pathway.

### Assessing combinations of pathways for enrichment

Above, we obtained evidence for enriched pathways in CD, RA and T1D. The question remains whether a combination of several enriched pathways offers a better fit to the data. A benefit of our approach is that we can compare support for enrichment of different combinations of pathways by comparing their BFs (assuming the same prior for these enrichment hypotheses).

**Figure 5:**
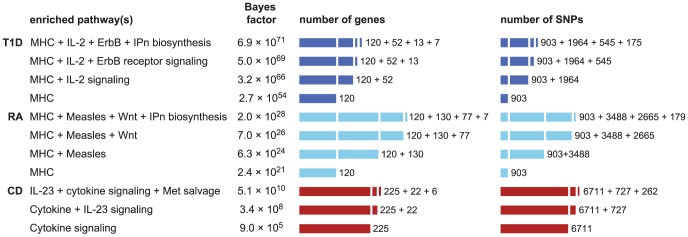
Enrichment hypotheses with multiple enriched pathways show increased support from data. Each row gives pathway, or combination of 2 or 3 pathways, with largest BF for enrichment of disease associations. See Figure 1 for legend and abbreviations used. All enrichment hypotheses for RA and T1D shown here also include enrichment of the MHC, allowing for a different level of enrichment within the MHC. Unlike the BFs in Figures 1 and 2, BFs here are all defined relative to null hypothesis of no enrichment, so that they can be easily compared. Counts of genes and SNPs only include those that are not already assigned to other enriched pathways; for example, 37 genes belong to the IL-23 pathway, and of those 15 are already cytokine signaling genes, so inclusion of IL-23 signaling adds 22 more genes. Databases and database identifiers for pathways in this figure: “IL2-mediated signaling events” (PID il2_1pathway, BS 137976); “ErbB receptor signaling network” (PID erbb_network_pathway, BS 138016); “Inositol pyrophosphates biosynthesis” (HumanCyc 6369, PC); “Measles” (KEGG hsa05162, BS 213306); “Wnt” (Cancer Cell Map, PC); “Cytokine signaling in immune system” (Reactome 75790, BS 366171); “IL23-mediated signaling events” (PID il23pathway, BS 138000); “Methionine salvage pathway” (Reactome 75881, BS 366245).

We assess support for combinations of pathways in CD, RA and T1D by computing BFs for models in which 2 and 3 pathways are enriched. Since it is impractical to consider all combinations of 2 and 3 pathways, we tackle this in a “greedy” fashion by selecting combinations of pathways based on the initial ranking (see Methods). Figure 5 gives the combinations of 2 and 3 pathways that yield the largest BFs for these diseases. Again, to properly interpret these results we must weigh these BF gains against the relative prior plausibility of the models. Using a “conservative” prior for any pair of pathways being enriched (see “Interpretation of Bayes factors” in Methods), we interpret Figure 5 as providing considerable, if short of compelling, support for the hypothesis that 2 pathways are enriched for disease associations in CD, RA and T1D. For example, in CD the BF for enrichment of both cytokine signaling and IL-23 signaling genes is 377 times greater than the BF for enrichment of cytokine signaling genes alone. The BFs for models in which 3 pathways show further increases, but not enough to constitute strong evidence for enrichment of 3 pathways.

We also examine whether enrichment of multiple pathways can lead to identification of additional loci affecting susceptibility to disease. Figure S3 shows that allowing for 2 enriched pathways in CD, RA and T1D does not yield strong support for associations beyond what is already revealed by enrichment of the single top pathway. We do, however, find that a segment near *IL12B* shows a substantial gain in support for association with CD (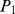 increases from 0.03 to 0.44), and this association is confirmed by other GWAS [6], [7], [132], [133].

## Discussion

Motivated by the observation that it is easier, at least in principle, to identify associations within an enriched pathway, we developed a data-driven approach to simultaneously assess support for enrichment of disease associations in pathways, and prioritize variants in enriched pathways. We investigated the merits and limitations of this approach in a detailed analysis of data sets for seven complex diseases. We interrogated thousands of candidate pathways from multiple pathway databases, finding strong evidence linking pathways to pathogenesis of several diseases. By promoting variants within the enriched pathways identified in our analysis, we mapped disease susceptibility loci beyond those identified by a conventional analysis.

The CD and RA results provided some validation for our methods, as all but one of the additional disease associations identified by pathway prioritization are corroborated by other studies. The T1D results also provided some validation for our methods, as several of the strongest associations informed by enrichment of the IL-2 signaling pathway are confirmed in other GWAS for T1D. Prioritizing IL-2 signaling genes revealed other regions relevant to T1D that could not be corroborated by other GWAS, and this may be because the largest GWAS for T1D to date does not match the scale of the largest studies for CD and RA. All the disease associations informed by enriched pathways had smaller effects on disease susceptibility, illustrating how pathway prioritization can help overcome some of the constraints on our ability to reliably detect disease-conferring variants with small effects in GWAS.

Our approach builds on methods that use multi-marker models to simultaneously map associated variants in GWAS [61], [75], [76], [134]–[142]. In contrast to single-marker regression approaches, these methods model susceptibility to disease by the combined effect of multiple variants, and use sparse multivariate regression techniques to fit multi-marker (*i.e.* polygenic) models to the data. Within a multi-marker model of disease, estimating enrichment of a candidate pathway effectively reduces to counting, inside and outside the pathway, variants associated with disease (more precisely, variants included in the multi-marker model). Our approach to combining multi-marker modeling with pathway analysis offers several benefits. First, unlike many pathway analysis methods that test for enrichment of significant SNPs or genes within a pathway [24], [25], we have no need to select a threshold to determine which *p*-values are significant; instead, we use the association signal from all variants to assess enrichment. Second, by analyzing variants simultaneously, we avoid exaggerating evidence for enrichment from disease-associated variants that are correlated with each other (*i.e.* in linkage disequilibrium), while still allowing multiple independent association signals near a gene to contribute evidence for enrichment. Third, and most importantly, quantifying enrichment within this framework gives us feedback about associations within enriched pathways, potentially leading to discovery of novel genetic loci underlying disease.

In contrast to many pathway analysis methods, we modeled enrichment of disease associations at the level of variants, rather than genes. While there are arguments for both approaches, a feature of the variant-based approach is that, when there are multiple variants near a gene that affect disease susceptibility, all these signals contribute to the evidence for enrichment of pathways containing this gene.

Another important feature of our approach is that it can be used to assess models in which multiple pathways are enriched. Examining combinations of pathways for enrichment may highlight pathways that would otherwise not be highly ranked. The results on RA and T1D provided vivid examples of this; evidence for enrichment of the Measles and IL-2 pathways only became compelling once we assessed support for enrichment of these pathways together with enrichment of the MHC.

Our results focused on the regions showing the strongest evidence for association with disease. However, the large number of points approaching the middle of the vertical axes in Figure 3 suggests that many other gene variants in the enriched pathways may contribute to risk of CD, RA and T1D; from our estimates of 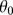 and 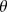 (Figure 2), approximately 38, 45 and 59 independent risk variants are, in expectation, hidden among Measles, cytokine signaling and IL-2 signaling genes, respectively. This suggests that more disease associations in these pathways remain to be discovered.

Several selected disease susceptibility regions (Table 1) contain multiple candidate genes, including cases in which the gene in the enriched pathway is not the same as the most credible gene suggested in prior studies. It is possible that pathway annotations would be useful to help pinpoint, or *fine-map*, the genes or variants relevant to disease within these regions. However, investigating this would require advances to our current methodology, as the approximations we made to improve the efficiency of our approach, building on earlier work [75], are less appropriate for refining the location of association signals, and these approximations will need to be modified to accommodate this aim. Nonetheless, we note that some of these regions may contain multiple variants that disrupt or regulate genes relevant to disease, and our methods can help assess this possibility. For example, we calculate that multiple independent risk variants reside at the 16p13 locus with probability 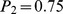 (Table 1). So it is possible that both *C1QTNF6* and *IL2RB* at this locus are associated with T1D risk variants.

A limitation of our current approach is that the prior variance of additive effects on disease risk must be chosen beforehand. We based our choice on the distribution of odds ratios reported in published genome-wide association studies, and checked that the ranking of enriched pathways was robust to different prior choices (see Methods). One problem with this prior is that published associations typically have the largest effects on disease risk, as these are the associations we usually have adequate power to identify. This results in a prior that places too much weight on larger additive effects. It would be preferable to estimate this prior from the data instead, but we found that this worked poorly in practice. The likely cause of this problem is that the non-zero effects on disease are not normally distributed, contrary to our assumptions. One possible solution would be to use a more flexible prior that is better able to capture the distribution of additive effects, such as a mixture of two or more normals [80].

In summary, our results on a range of complex diseases illustrate how an integrated approach to identification of enriched pathways, and prioritization of variants within enriched pathways, can identify additional disease associations beyond standard statistical procedures based on single-marker regression. Our results point to the potential for applying our methods to other common diseases, and larger studies, to uncover genetic loci that have not yet been identified as risk factors for disease.

## Methods

### Samples

Results on all seven diseases are based on genome-wide marker data from the case-control studies described in the original WTCCC study [65]. For all diseases, the control samples come from two groups: 1480 individuals from the 1958 Birth Cohort (58BC), and 1458 individuals from the UK Blood Services (UKBS) cohort. All subjects are from Great Britain, and are of self-described European descent. Genetic associations from these studies were first reported in [65].

All study subjects were genotyped for roughly 500,000 SNPs on autosomal chromosomes using a commercial version of the Affymetrix GeneChip 500K platform. We estimate missing genotypes at the SNPs using the mean posterior minor allele count from BIMBAM [79], [143], with SNP data from Phase II of the International HapMap Consortium project [144]. To be consistent with the original analysis, refSNP identifiers and locations of SNPs are based on Human Genome Reference Assembly 17 (NCBI build 35).

We apply quality control filters as described in [65], and remove SNPs that exhibit no variation in the sample. For all diseases, we include an additional quality control measure to filter out potentially problematic SNPs. Some SNPs with high minor allele frequencies (MAFs) show moderate evidence for association based on our calculations—“single-SNP” BFs [79] in which the prior standard deviation of the log-odds ratios is set to 0.1—but because they do not appear to be supported by nearby SNPs upon inspecting their single-SNP BFs, we cannot rule out the possibility of genotyping errors. Based on this criterion, we discard 2 additional SNPs in CD, rs1914328 on chromosome 8 at 69.45 Mb (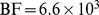, MAF = 0.43), and rs6601764 on chromosome 10 at 3.85 Mb (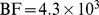, MAF = 0.43). In each case, no nearby SNPs have single-SNP BFs greater than 46. For CAD, we discard SNP rs6553488 on chromosome 4 at 171.4 Mb (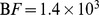, MAF = 0.46). No nearby SNPs have a single-SNP BF greater than 11. Following the same quality control criterion, we do not filter out SNPs in the other data sets. Table S1 summarizes the data used in our analysis after following these quality control steps.

### Pathways, and assignment of SNPs to genes in pathways

**Figure 6:**
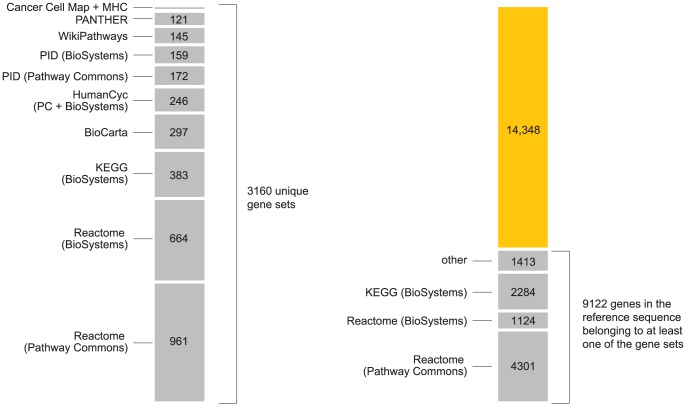
Summary of pathways used in the analysis. Chart on left shows number of unique gene sets obtained from the following pathway databases, included in this order: Reactome [167], Kyoto Encyclopedia of Genes and Genomes (KEGG) [146], BioCarta (www.biocarta.com), HumanCyc [147], [168], NCI Nature Pathway Interaction Database (PID) [163], WikiPathways [169], [170], PANTHER [171] and Cancer Cell Map (cancer.cellmap.org). The majority of these pathways are retrieved from the Pathway Commons (PC) [165] and NCBI BioSystems [164] repositories. We include gene sets from both repositories when gene sets from same pathway differ (see Supplementary Materials). We include two additional gene sets for “classical” and “extended” MHC [90], [91]. Right-hand chart shows gains in gene coverage by including additional databases in the analysis, where “gene coverage” is defined as any genes in reference sequence that are assigned to at least one pathway. From the total of 3160 gene sets (including MHC and ×MHC), we achieve coverage of 39% of genes in reference sequence (see Figure S6).

We aim for a comprehensive evaluation of pathways accessible on the Web in standard, computer-readable formats [63], [64], [145]. Since the results hinge on the quality of the pathways used in the analysis, we restrict the analysis to curated, peer-reviewed pathways based on experimental evidence, and pathways inferred via gene homology. We draw candidate pathways from the collections listed in Figure 6 (see also Supplementary Materials). KEGG [146] and HumanCyc [147] are primarily databases of metabolic pathways, and are unlikely to be relevant to some autoimmune diseases, but for completeness we include them in the analysis of all diseases. We create 2 additional gene sets to assess support for enrichment of disease associations within the MHC and “extended” MHC (xMHC) [90], [91]. We treat each candidate pathway as a set of genes, ignoring details such as molecules involved in biochemical reactions, and cellular locations of these reactions.

Many pathways are arranged hierarchically in the databases; we include all elements of the hierarchy in our analysis. Elements in upper levels of the hierarchy refer to groups of pathways with shared attributes, or a common function. Some gene groups have a broad definition, such as “Immune system” in Reactome (ID 6900), which includes pathways involved in adaptive and innate immune response. Enrichment of a large gene set is unlikely to provide much insight into disease pathogenesis. However, a key step in our analysis is to re-interrogate SNPs for association in light of inferred enrichments. Thus, enrichment of a broad physiological target such as “Immune system” can be useful if subsequent re-interrogation reveals associations that were not significant in a conventional analysis.

Since we combine pathways from different sources, we encounter pathways with inconsistent definitions [148], [149]; see Supplementary Materials. There is no single explanation for this lack of consensus, and we have no reason to prefer one definition over another, so we include all versions of pathways in our analysis.

Based on findings that the majority of variants modulating gene expression lie within 100 kb of the gene's transcribed region [150]–[152], we assign a SNP to a gene if it is within 100 kb of the transcribed region. Others have opted for a 20 kb window [69], [73] based on findings that *cis*-acting expression QTLs are rarely more than 20 kb from the gene [62]. We choose a broader region since the benefit of including potentially relevant SNPs in a pathway when the association signal is sparse seems likely to outweigh the cost of including a larger number of irrelevant markers.

### Selection of combinations of pathways

In our case studies on CD, RA and T1D, we compute BFs to assess support for models in which 2 and 3 pathways are enriched for disease associations. Since it is impractical to consider all combinations of 2 and 3 pathways, we tackle this in a “greedy” fashion by selecting combinations of pathways based on the initial ranking. Our strategy is to select the pathway with the largest BF (Figure 1), and assess support for this pathway in combination with pathways from a larger set of candidates (we take all pathways with BF>10). This heuristic makes it feasible to evaluate many combinations of pathways that could plausibly be jointly enriched, though it does not consider all combinations, so we may miss a combination with stronger evidence for enrichment. In total, we compute BFs for 85, 24 and 408 pairs of pathways in CD, RA and T1D, respectively. (Note that models for RA and T1D also include enrichment of the MHC.) For completeness, we extend the analysis to models with 3 enriched pathways. Following the same greedy strategy, we take the top pair of pathways (Figure 5) and combine it with individual pathways with BF>10.

### Statistical analysis

The Bayesian variable selection approach to simultaneous interrogation of SNPs involves fitting a multi-marker disease model to the data with different combinations of SNPs. By accounting for correlations between markers, fitting all markers simultaneously allows us to identify those that are *independently associated*—that is, markers that individually signal a variant contributing to disease risk independently of other risk-conferring variants.

#### Likelihood

The likelihood specifies the probability of observing disease (case-control) status *y* given the genotypes 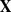, the intercept 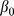, and the regression coefficients 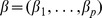. From the additive model for the log-odds of disease (eq. 1), 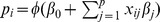 is the probability that 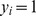, in which 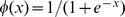 is the sigmoid function. Assuming independence of the observations 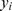, the likelihood is
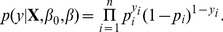
(4)


#### Priors

Next we specify prior distributions for the following model parameters: the genome-wide log-odds 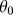, the log-fold enrichment 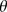, the intercept 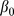, and the coefficients 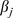 of included SNPs.

Since inferences strongly depend on 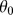, and since 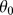 is unknown and will be different for each setting, we estimate this parameter from the data. Following [75], [76], we assign a uniform prior to 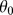. We restrict 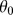 to 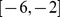, so as few as 0 and as many as ∼4400 SNPs are expected to be included *a priori*. We assign a uniform prior to 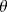 on interval 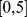. This allows for a wide range of enrichments.

For the prior on the non-zero coefficients 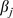, we follow standard practice that assumes they are *i.i.d.* normal with zero mean and standard deviation 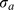 [153]. Ordinarily, to combat sensitivity of the results to the choice of 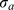, we would place a prior on 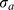 and integrate over this parameter to let the data drive selection of 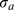. This approach is taken in [75], [76]. But in our case we find that the heterogeneity of the odds ratios in complex diseases presents a problem: although we expect most odds ratios for a common disease—and specifically odds ratios in a pathway relevant to disease pathogenesis—to be close to 1, the odds ratios corresponding to the strongest disease associations drive estimates of 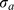 toward larger values, and a normal distribution that puts too little weight on modest odds ratios. One possible strategy would be to redo the analysis after removing associated regions with the largest odds ratios, but this is an unattractive solution because SNPs with large odds ratios would not contribute to the evidence for enrichment. Instead, we fix 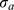, grounding the choice on typical odds ratios reported in published GWAS, and we assess the robustness of our findings to this choice. Our choice is 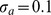, which favours odds ratios close to 1 (95% of the odds ratios lie between 0.82 and 1.22 *a priori*), while being large enough to capture a significant fraction of the odds ratios for common alleles reported in genome-wide association studies of complex disease traits. According to a recent review [154], approximately 40% of estimated odds ratios are between 1.1 and 1.2, and an additional 10% of odds ratios are smaller than 1.1. This prior also closely corresponds to a survey of odds ratios reported in genetic association studies of common diseases [155]. Since there may be justification for a slightly smaller or slightly larger 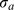, we also try different values for 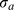, and examine how these choices affect the ranking of enriched pathways in the CD data set (see below).

To complete the probability model, we assign an improper uniform prior to the intercept, 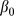. In general, one must be careful with use of improper priors in Bayesian variable selection because they can result in improper posteriors. A sufficient condition for a proper posterior, and a well-defined BF, with logistic regression (eq. 1) is that the maximum likelihood estimator of *β* conditioned on which variables are included in the model, and on the other model parameters, is unique and finite [156]. This condition is difficult to check exhaustively in Bayesian variable selection, but we can at least guarantee that the posterior is proper under the variational approximation (see Supplementary Materials) so long as the coordinate ascent steps converge to a unique solution.

#### Sensitivity of pathway ranking to prior distribution of odds ratios

**Figure 7:**
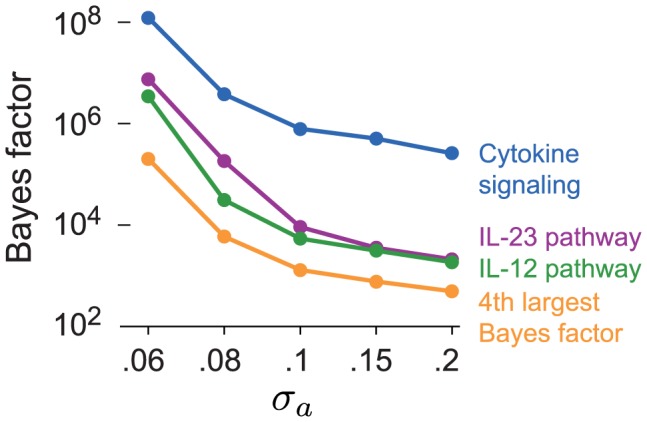
Top four BFs in CD for each setting of 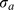. In each case, the 3 largest BFs correspond, in order, to Cytokine signaling in immune system, IL23-mediated signaling events, and IL12-mediated signaling events (these are the top 3 pathways for CD in Figure 2). Pathway with fourth largest BF differs across settings of 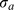.

A concern with our choice of prior for *β* is that slightly smaller or slightly larger settings of 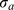 could also be justified, and these choices could produce different results. Associations are unlikely to accumulate at a greater rate in pathways that are not related to the disease, even associations with small effects on disease risk, so we predict that the ranking of enriched pathways is largely robust to 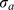. Here we verify this claim on the CD data set. We assess the sensitivity to 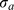 by recomputing the BFs for all candidate pathways with prior choices that favor slightly smaller (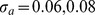) and slightly larger coefficients (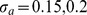). Figure 7 shows that smaller settings of 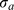 yield substantially more support for enrichment of CD-related pathways, as expected. But the pathways with the largest BFs remain IL-23, IL-12 and cytokine signaling regardless of the choice of 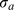. In Supplementary Materials, we show that the BFs for most other candidate pathways do not change noticeably at different settings of 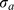.

#### Bayes factors

We adopt the *Bayesian model averaging* strategy [82], [135], [157] to account for possible uncertainty in 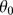 and 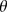 when evaluating the BFs (eq. 3). The likelihood under the enrichment hypothesis (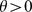) and the likelihood under the null (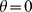) are each expressed as an average over possible assignments to 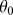 and 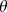:
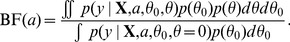
(5)Each instance of 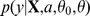 in (5) expands as an average over possible assignments to the intercept 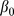 and regression coefficients *β*:
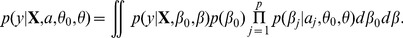
(6)The factors in this equation are all determined by the model and priors specified above. Factor 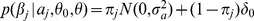 is the “spike and slab” prior [153], [158], in which 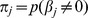 is given by eq. 2. Here, 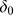 denotes the delta mass, or “spike”, at zero, and 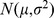 is the normal density with mean 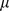 and variance 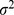. Factor 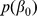 is the (improper) uniform prior, and 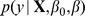 is the logistic regression likelihood (4). Computation of the BFs is described in the Supplementary Materials.

#### Interpretation of Bayes factors

Given that enrichment analyses typically proceed by computing *p*-values and assessing “significance,” one may reasonably ask whether a given BF represents “significant” evidence for enrichment. Specifying an appropriate threshold for a BF to be considered significant, however, is context-dependent, and subjective. This is because the *posterior odds* for a pathway being enriched, relative to the null hypothesis that no pathways are enriched, is equal to the BF times the prior odds for enrichment, and the prior odds for each pathway depends on how plausible it is, *a priori*, that the pathway is relevant to the disease. (Similar issues arise when specifying significance thresholds for *p*-values; for example, the false discovery rate at a given *p*-value threshold depends on the prior probability of enrichment [159], [160]. But in practice significance thresholds of 0.01 or 0.05 are often used without attending to this concern.) Nonetheless, we can make the following observations. First, if we are willing to assume the pathways in Figures 1 and 2 are all equally plausible candidates for enrichment *a priori*, then the ratio of BFs indicates the relative support for the enrichment hypotheses; for example, if we must choose between enrichment of cytokine signaling genes and the IL-23 signaling pathway, the data overwhelmingly favour the former by a factor of 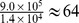 (Figure 2). Second, even under a “conservative” prior for enrichment in which we expect 1 pathway to be enriched among the 3158 candidates, corresponding to a prior odds of 1/3158, the top pathways in CD and T1D are large enough to strongly support enrichment (the BFs are all much greater than 3158). Weighing this prior against the BF for the top pathway in RA does not yield strong support for this pathway, but given its plausible connection to RA, we view this enrichment result as compelling.

We can apply similar reasoning to weigh the evidence for hypotheses in which 2 or more pathways are enriched. For example, the model in which both cytokine signaling and IL-23 pathway genes are enriched for CD associations has a BF that is ∼400 times greater than the BF for enrichment of cytokine signaling genes alone. This indicates that the best model with 2 enriched pathways provides a much better fit to the CD data than the best model with any one enriched pathway. However, to properly interpret this result we must weigh this increase in the BF against the relative prior plausibility of the models. A naive argument using a “conservative” prior for any pair of pathways being enriched might suggest a prior odds of 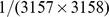. This prior would make a 400-fold increase in the BF appear to be relatively insignificant. However, this argument not only depends on the earlier prior, which may be overly conservative, but also assumes independence of enriched pathways, which seems unwise considering many pathways mentioned in the results have related roles in immunity; *a priori*, one might expect that a pathway is more likely to be enriched when a related pathway is enriched.

#### Posterior inclusion probabilities and other posterior quantities

In this section, we define posterior inclusion probabilities (PIPs) and other posterior quantities used in the results. In all cases, posterior statistics under the null hypothesis are obtained by setting 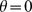.

Like the BFs, the PIPs are obtained by averaging over 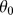 and 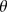. Taking 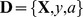 as shorthand for the GWAS data, we have
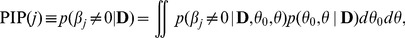
(7)where 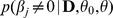 is the PIP for marker *j* given hyperparameter setting 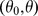, and 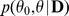 is the posterior probability of hyperparameter setting 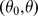. Computation of these posterior probabilities is described in Supplementary Materials.

To identify regions of the genome associated with disease risk, for each region we calculate the posterior probability that at least 1 SNP in the region is included in the multi-marker disease model. Let 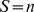 represent the event that exactly *n* SNPs in a given region are included in the multi-marker disease model, so that 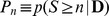. These posterior probabilities are easily calculated from the PIPs (7) using the variational approximation; see Supplementary Materials.

Since no single pathway stands out in Figure 2 as having greatest support for enrichment of T2D associations, we compute posterior quantities by averaging over different enrichment models with the largest BFs, weighting these models by their BFs. We do the same for models in which 2 or 3 pathways are enriched. (Implicitly, this assumes all models are equally plausible *a priori*.) The ability to average across models in this way is an advantage the Bayesian model comparison approach, because it allows us to assess associations in light of the enrichment evidence without having to choose a single enrichment model. Suppose we have *m* enrichment models, specified by their SNP annotations 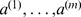, with corresponding BFs, 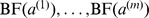. Then 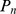 is given by
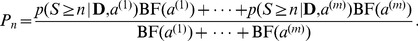
(8)See Supplementary Materials for further details about computation of relevant posterior quantities.

#### Population stratification

Pathway enrichment analysis should be robust to population stratification because spurious associations that arise from population structure are unlikely to accumulate at a greater rate in the pathway. Further, the original report [65] and subsequent analyses [6], [161] affirm that cryptic population structure does not have a substantive impact in these data. Thus we did not correct for population structure in our analysis.

#### Modifications to analysis to account for large contributions of MHC alleles to RA and T1D risk

Recent work by Pirinen *et al* [162] has shown that when analyzing a case-control study with a prospective model, as we do here, controlling for non-confounding covariates, particularly those with large effects, can reduce power to detect associations. Their work implies that joint modeling of the effects of multiple SNPs on disease risk could actually reduce power to detect associations compared with single-marker tests, particularly for diseases such as RA and T1D in which MHC variants are known to have large effects on disease risk. Here we briefly explain how we modify our approach to address this issue.

Pirinen *et al* [162] recommend omitting non-confounding covariates when the goal is to discover new loci associated with disease. SNPs in the MHC associated with disease are non-confounding covariates of large effect, so we could adhere to their advice simply by ignoring all MHC SNPs when analyzing the non-MHC SNPs. However, omitting the MHC would prevent us from effectively interrogating pathways that contain MHC genes. Instead, we develop the following two-stage procedure: first, we fit our model using only SNPs outside the extended MHC to estimate their effects, 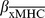; second, fixing 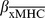 to the estimates obtained from the first step, we fit the multi-marker model to the full data to estimate 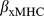, the effects of SNPs within the ×MHC. This procedure is equivalent to assuming that 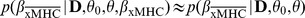. (See Supplementary Materials for details on how this assumption is incorporated into the variational approximation to efficiently compute BFs and posterior statistics.) Thus, in the first stage we ignore the MHC when deciding which non-MHC SNPs to include in the multi-marker model, following the advice from [162], but in the second stage we allow that the MHC may contribute evidence toward pathway enrichment when the pathway includes MHC genes.

We found that addressing these issues was important for analyzing the RA and T1D data. With these modifications, our results more closely replicate the original single-marker association analysis (Figures S4 and S5), and our methods yield more support for enrichment of pathways that overlap the MHC.

#### Analyses conditional on MHC enrichment

Once we establish that the MHC has far greater support for enrichment of disease associations in RA and T1D than any other candidate pathway, we condition on enrichment of the MHC, and search for gene sets with evidence for enrichment beyond the MHC. To speed up computation, we fix the MHC enrichment parameter, 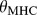, to its *maximum a posteriori* estimate, and we additionally assume that the posterior distribution of 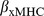 is unaffected by enrichment of the candidate pathway; that is, 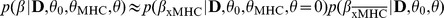. (Note that we make a similar approximation to improve efficiency of computation for all pathways. We assume that coefficients 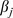 for all SNPs *j* outside the enriched pathway are unaffected by pathway enrichment *a posteriori*; see Supplementary Materials.) To assess support for enrichment of a candidate pathway in RA and T1D, we compare the likelihood given the model in which the candidate pathway and the MHC are enriched to the likelihood given the model in which only the MHC is enriched. Thus, unless otherwise specified, all BFs for enrichment of pathways in RA and T1D are defined relative to the “null” that the MHC is enriched, rather than the null of no enrichment.

### Software availability

MATLAB implementations of the statistical methods described here, and the MATLAB scripts used to implement the steps in our analysis, are available for download at http://github.com/pcarbo/bmapathway.

## Supporting Information

Figure S1More pathways and their BFs for enrichment of disease associations. Columns left to right: (1) enriched pathway; (2) BF for hypothesis that disease associations are enriched among SNPs assigned to pathway; (3) number of genes assigned to pathway; (4) number of SNPs near these genes; (5,6) posterior mean and 95% credible interval of genome-wide log-odds (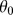) and log-fold enrichment (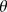) given that pathway is enriched (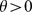). BFs for pathways marked by _*_ are conditioned on enrichment of disease associations in MHC, in which SNPs within MHC are enriched at a different level than non-MHC SNPs in pathway; 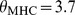 and 4.6 for RA and T1D, respectively. Number of genes and SNPs for RA and T1D show counts within MHC, and outside the MHC that are assigned to pathway. The BFs for these pathways are defined relative to null with no enrichment so that the BFs are directly comparable. Enrichment results for RA and T1D that do not condition on enrichment of MHC are indicated by 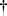. Database identifiers for pathways not previously mentioned: “Asthma” (KEGG hsa05310, BS 83120); “Allograft rejection” (KEGG hsa05330, BS 83123); “Cyclin D associated events in G1” (Reactome 821, BS 105767); “Signaling by interleukins” (Reactome 22232, BS 160140); “Interferon gamma signaling” (Reactome 25078, BS 187106); “S6K1-mediated signaling” (Reactome 6754, BS 106433); “Calcineurin-regulated NFAT-dependent transcription in lymphocytes” (PID nfat_tfpathway, BS 137993); “Validated transcriptional targets of AP1 family members Fra1 and Fra2” (PID fra_pathway, PC); “p38 MAPK signaling pathway” (PID p38_mkk3_6pathway, PC); “Regulation of p38-alpha and p38-beta” (PID p38alphabetapathway, PC); “Cell cycle: G1/S check point” (BioCarta); “Incretin synthesis, secretion, and inactivation” (Reactome, BS 187170); “Synthesis, secretion, and inactivation of glucagon-like Peptide-1” (Reactome, BS 187171). **Note that several pathways not shown in this figure have larger BFs in CD than the MHC and ×MHC.(EPS)

Figure S2 Distribution of BFs for enrichment in seven diseases. Panels with blue bars give the distribution of BFs compiled across all 3160 candidate pathways, including the MHC and ×MHC. The two lower-right panels with orange bars (*) each give the distribution of BFs for all candidate gene sets except the MHC and ×MHC, after conditioning on enrichment of the MHC.
(EPS)

Figure S3Scatterplots showing 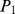 conditioned on enrichment hypotheses with 1, 2 and 3 enriched pathways in CD, RA and T1D. Each point corresponds to a contiguous segment of the genome containing 50 SNPs. Since no single combination of pathways stands out in BF-based rankings of 2 or 3 enriched pathways (results not shown), 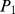 conditioned on enrichment of 2 or 3 pathways is obtained by averaging over different enrichment models with the largest BFs. Points highlighted in red correspond to segments overlapping SNPs assigned to at least one of the enriched pathways included the top enrichment hypotheses, excluding pathways that are already enriched in hypotheses shown in horizontal axis. For example, in top-left panel, a segment is highlighted in red if it overlaps SNPs assigned to pathways included in enrichment hypotheses with the largest BFs, excluding SNPs assigned to cytokine signaling genes. In RA and T1D, 50-SNP segments overlapping the MHC are shown as open circles (SNPs in these segments are not assigned to the pathways). Asterisk (*) indicates an appreciable increase in the probability of disease association, and this association is validated by other GWAS for same disease.(EPS)

Figure S4Comparison of single-marker and multi-marker mapping of disease associations without pathways. Each point corresponds to a region of the genome that shows moderate to strong support for disease risk factors based on our analysis using a multi-marker disease model, or based on testing each SNP separately for correlation with disease risk, following [65]. Precisely, points correspond to regions for which 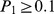 or for which smallest trend *p*-value in [65] is less than 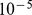. All regions to the right of 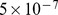 in the left-hand plot are listed in Table 3 of [65]. Horizontal axis in right-hand plot shows the logarithm of the “additive” Bayes factor from the single-marker analysis in [65], which correlates well with the trend *p*-value. All *p*-values less than 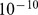 are shown as 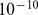, and all *p*-values greater than 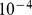 are shown as 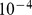. Similarly, Bayes factors are projected onto interval 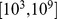. Points furthest away from diagonal are labeled by their location on the chromosome.(EPS)

Figure S5Comparison of multi-marker mapping in RA and T1D with and without MHC-related modifications. Horizontal axis shows 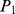 when all SNPs across the genome are analyzed jointly, and vertical axis shows 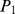 when SNPs outside the MHC are analyzed separately in the first step (see Methods). Each point corresponds to a segment of the genome containing 50 SNPs. Segments overlapping the extended MHC are shown in light blue. Points furthest away from diagonal are labeled by their location on the chromosome unless they overlap with the MHC. In RA and T1D, we obtain strong support for multiple disease risk variants mapping to the MHC.(EPS)

Figure S6 Pathway database statistics. *Panel A:* gene set sizes. *Panel B:* sizes of SNP annotations corresponding to gene sets. *Panel C:* number of pathways assigned to each gene. *Panel D:* number of pathway annotations for each SNP. SNP counts are based on CD data set. Pathway counts include multiple versions of the same pathway drawn from Pathway Commons and NCBI BioSystems repositories.
(EPS)

Figure S7Posterior estimates of 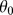 under null. Mean and 95% credible interval of 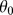 for each disease under null hypothesis that no pathways are enriched. Filled circles show posterior means. Error bars depict 95% credible intervals.(EPS)

Figure S8Genome-wide scans without pathways for BD, CAD, CD and HT. Each point corresponds to a 50-SNP segment of the genome. Height of each point gives 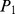, the posterior probability that at least one SNP in the segment is included in the multi-marker disease model. Segments are ordered by chromosome, then by position along chromosome. Autosomal chromosomes 1–22 are shown in alternating shades of blue. Non-overlapping segments with 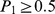 are listed in Table S2.(EPS)

Figure S9 Genome-wide scans without pathways for RA, T1D and T2D. See Figure S8 for an explanation of this figure.
(EPS)

Figure S10Comparison of BFs for enrichment using coarse and finely spaced grids. Scatterplot shows numerical estimates of BFs for enrichment of disease associations using an grid with wide intervals for 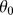 and 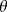 (horizontal axis), and numerical estimates of the same BFs using a grid spaced at intervals of 0.1. BFs for RA and T1D include enrichment of the MHC. Omitted from this plot are BFs for RA and T1D that do not include enrichment of the MHC.(EPS)

Figure S11Posterior mean of 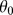 estimated from CD data given different settings of 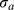. Error bars depict 95% credible intervals.(EPS)

Figure S12Distribution of BFs in CD given different settings of 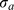. The top 4 BFs for each setting of 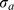 are shown in Figure 7.(EPS)

Table S1 Summary of data from WTCCC studies.
(PDF)

Table S2 Regions of the genome with moderate to strong evidence for disease risk factors under null.
(PDF)

Text S1 Supplementary text containing more results and additional details about methods.
(PDF)
