# Supplementary material for: Integrated Enrichment Analysis of Variants and Pathways in Genome-Wide Association Studies Indicates Central Role for IL-2 Signaling Genes in Type 1 Diabetes, and Cytokine Signaling Genes in Crohn's Disease
Source: PLoS Genet. 2013 Oct 3;9(10):e1003770. doi: 10.1371/journal.pgen.1003770 (PMC3789883; doi:10.1371/journal.pgen.1003770)
Supplement: Table S1 — Summary of data from WTCCC studies. (PDF) [file pgen.1003770.s013.pdf]

Table S1. Summary of data from WTCCC studies.

disease	bipolar disorder	coronary artery disease	Crohn's disease	hyper-tension	rheumatoid arthritis	type 1 diabetes	type 2 diabetes
cases	1868	1926	1748	1952	1860	1963	1924
controls	2938	2938	2938	2938	2938	2938	2938
SNPs	441,283	440,807	442,001	441,089	441,079	441,627	441,398

The bottom row shows the total number of SNPs for each disease after applying quality control filters, discarding additional SNPs that may exhibit genotyping errors, and after removing SNPs that show no variation in the sample (see Methods).
