# Supplementary material for: Integrated Enrichment Analysis of Variants and Pathways in Genome-Wide Association Studies Indicates Central Role for IL-2 Signaling Genes in Type 1 Diabetes, and Cytokine Signaling Genes in Crohn's Disease
Source: PLoS Genet. 2013 Oct 3;9(10):e1003770. doi: 10.1371/journal.pgen.1003770 (PMC3789883; doi:10.1371/journal.pgen.1003770)
Supplement: Table S2 — Regions of the genome with moderate to strong evidence for disease risk factors under null. (PDF) [file pgen.1003770.s014.pdf]

Table S2. Regions of the genome with moderate to strong evidence for disease risk factors under null.

disease	chr.	region (Mb)	P_1	P_2	candidate gene(s)	SNP	PIP	LOR (95% CI)	MAF ctrls	MAF cases
CAD	9p21	21.82–22.12	1.00	0.00	*CDKN2B*	rs9632884	1.00	-0.26 (0.19–0.33)	0.522	0.445
CD	1p31	67.31–67.57	1.00	0.06	*IL23R*	**rs11805303**	1.00	0.25 (0.18–0.33)	0.318	0.391
CD	2q37	233.74–234.04	1.00	0.01	*ATG16L1*	**rs10210302**	1.00	-0.27 (0.21–0.36)	0.481	0.402
CD	5p13	40.24–40.53	1.00	0.43	*PTGER4*	**rs17234657**	1.00	0.29 (0.20–0.39)	0.124	0.181
CD	6	MHC	0.51	0.02	multiple	rs9469220	0.48	-0.17 (0.10–0.25)	0.519	0.465
CD	10q21	64.08–64.27	0.96	0.03	*ZNF365*	rs10995271	0.96	0.20 (0.13–0.28)	0.386	0.440
CD	10q24	101.24–101.38	0.95	0.01	*NKX2-3*	rs7095491	0.95	0.20 (0.12–0.27)	0.470	0.527
CD	16q12	49.11–49.42	1.00	0.11	*NOD2*	**rs17221417**	1.00	0.24 (0.17–0.32)	0.287	0.356
CD	18p11	12.48–12.99	0.94	0.01	*PTPN2*	**rs2542151**	0.94	0.24 (0.14–0.32)	0.163	0.209
RA	1p13	113.85–114.32	1.00	0.00	*PTPN22*	**rs6679677**	1.00	0.49 (0.39–0.60)	0.096	0.169
RA	6	MHC	1.00	1.00	multiple	rs9268560	1.00	-0.38 (0.31–0.46)	0.483	0.306
T1D	1p13	113.71–114.26	1.00	0.01	*PTPN22*	**rs6679677**	1.00	0.51 (0.40–0.61)	0.096	0.170
T1D	6	MHC	1.00	1.00	multiple	rs9273363	1.00	0.80 (0.72–0.87)	0.305	0.709
T1D	12q13	54.36–54.97	0.94	0.00	*ERBB3*	rs1873914	0.94	0.22 (0.14–0.29)	0.414	0.471
T1D	12q24	110.65–111.28	1.00	0.00	*SH2B3*	**rs17696736**	1.00	0.32 (0.24–0.39)	0.424	0.505
T1D	16p13	11.04–11.28	0.51	0.01	*CLEC16A*	**rs12708716**	0.51	-0.20 (0.13–0.29)	0.350	0.297
T2D	10q25	114.58–115.00	1.00	0.01	*TCF7L2*	rs7901695	1.00	0.26 (0.19–0.34)	0.321	0.391
T2D	16q12	52.04–52.38	0.77	0.01	*FTO*	rs9939973	0.51	0.14 (0.01–0.24)	0.428	0.481

For each region in this table, there is at least a 0.5 probability that one or more SNPs in the region is included in the multi-marker disease model ($P_1 \geq 0.5$) under the null hypothesis that no pathways are enriched for disease associations. Each region is a segment containing 50 SNPs. Overlapping segments containing the same association signal are not included in this table. Table columns from left to right are: (1) disease; (2) chromosomal locus; (3) region of the genome spanned by the 50 SNPs, in Megabases; (4) posterior probability that one or more SNPs in the segment are included in the model under the null hypothesis; (5) posterior probability that 2 or more SNPs are included under the null; (6) established genes in disease pathogenesis, or most credible genes of interest, corresponding to the locus; (7) refSNP identifier of SNP in segment with largest PIP (SNP in bold corresponds exactly to SNP in [65] with the smallest p-value); (8) the PIP of that SNP; (9) posterior mean and 95% credible interval of regression coefficient β_j, or equivalently additive effect of minor allele count on log-odds of disease ("log-odds ratio"), in multi-marker disease model conditioned on SNP being included in model; (10) frequency of minor allele for that SNP in controls, and (11) in cases. All SNP information and genomic positions are based on Human Genome Assembly 17 (NCBI build 35).
