# Supplementary material for: Integrated Enrichment Analysis of Variants and Pathways in Genome-Wide Association Studies Indicates Central Role for IL-2 Signaling Genes in Type 1 Diabetes, and Cytokine Signaling Genes in Crohn's Disease
Source: PLoS Genet. 2013 Oct 3;9(10):e1003770. doi: 10.1371/journal.pgen.1003770 (PMC3789883; doi:10.1371/journal.pgen.1003770)
Supplement: Text S1 — Supplementary text containing more results and additional details about methods. (PDF) [file pgen.1003770.s015.pdf]

Supplementary text for “Integrated enrichment analysis of variants and pathways in genome-wide association studies indicates central role for IL-2 signaling genes in type 1 diabetes, and cytokine signaling genes in Crohn’s disease”

Peter Carbonetto[1,*], Matthew Stephens[1,2]
1 Dept. of Human Genetics, University of Chicago, Chicago, IL, USA
2 Dept. of Statistics, University of Chicago, Chicago, IL, USA
* E-mail: pcarbo@uchicago.edu

Retrieval of gene sets from pathway databases **1**

Computation for statistical analysis **2**
Computing the Bayes factor for one pathway 3
Computing the posterior inclusion probabilities and other posterior statistics 4
Scaling computation to many pathways, and modifications to computation to account for large contributions of MHC alleles to RA and T1D risk 5

More details on analysis of seven common diseases **6**
Multi-marker disease mapping under null model 7
Bayes factors for enriched gene sets 8
Bayes factors for combinations of enriched pathways 9
Associations informed by enriched pathways 9
Associations informed by multiple enriched pathways 10
Additional results from sensitivity analysis 10

Retrieval of gene sets from pathway databases

We retrieve most pathway definitions from the Pathway Commons [165] and NCBI BioSystems [164] repositories. From the Pathway Commons website, we download the October 26, 2011 version of Gene Matrix Transposed `(.gmt)` file for *homo sapiens*. This file includes gene sets derived from Cancer Cell Map, HumanCyc, PID and Reactome pathways. To retrieve BioSystems pathways, we first get pathway names and IDs by searching for `“homo sapiens”[organism]`, then save the search result as a CSV file. Next, we download the November 15, 2011 version of the `biosystems_gene` file from the NCBI FTP site, which provides associations between genes and pathways. The BioSystems database contains pathways from HumanCyc, KEGG, PID, Reactome and WikiPathways.

Pathway Commons and NCBI BioSystems include pathways from the same sources, but due to differences in versions of the databases and data processing procedures, we observe discrepancies among pathways. As of this writing, BioSystems ignores nesting relationships between pathways in the PID. This can lead to disagreements in pathway gene sets between Pathway Commons and BioSystems, and differences in BFs for enrichment of disease associations.[1] For example, the BF for the Pathway Commons version of “Cytokine signaling in immune system” is smaller than the BioSystems version of the same pathway in CD by a factor of over 1000, due primarily to the lack of inclusion of *NOD2* and HLA genes that contribute to the evidence for enrichment. Conversely, the BF for the Pathway Commons version of “IL23-mediated signaling events” is about 80 times larger than the BioSystems version of the same pathway. The Pathway Commons version includes the NF-kB pathway, which contains several genes, notably *NOD2*, that contribute evidence for enrichment. (It may be helpful to know that inclusion of the NF-kB pathway is supported by experimental evidence [187].) Since we cannot account for all discrepancies in

[1]Personal communication with Lewis Geer and Emek Demir.

BioSystems and Pathway Commons gene sets, whenever there is disagreement we include both gene sets in our analysis, and we assess evidence for enrichment of these gene sets separately.

We download a version of the BioCarta database at `www.openbioinformatics/gengen`. We use this version of the BioCarta data because it was used in an previous pathway analysis of CD [73].

We download version 3.01 of the PANTHER "sequence association" file from the FTP site, following the link at `www.pantherdb.org/pathway`. From the sequence association file, we retain lines containing `ENSG*` accession numbers (corresponding to human genes), and we remove entries that do not map to Entrez gene IDs.

We create two additional gene sets to assess support for enrichment of disease associations within the MHC. The first gene set is defined as the 120 genes within the "classical" MHC that are known to be expressed [90]. This annotation includes all SNPs located in the 3.6 Mb region between the gene closest to the telomere, *HLA-F*, and the gene closest to the centromere, *HLA-DPB1*, on 6p. The other gene set is defined as the 244 expressed gene loci within the 7.6 Mb "extended" MHC region (xMHC) [91]. This annotation includes all SNPs between *HIST1H2AA* and *KIFC1*.

Finally, we discard 213 pathways with less than two genes in the reference genome, and we remove 44 PID pathways from Pathway Commons that contain over 500 genes because their definitions include a large number of nested pathways. We include all groups of related pathways except for two unusually large gene sets from KEGG, "Metabolic pathways" (hsa01100) and "Pathways in cancer" (hsa05200).

We observe high variation in the number of genes and SNPs assigned to each pathway (Figure S6, Panels A and B). Some of the larger gene sets are groups of related pathways in the Reactome and PID pathway hierarchies. The number of pathways assigned to each gene also varies widely (Figure S6, Panel C). Out of approximately 23,500 genes in the reference genome, 9122 (39%) are assigned to at least one pathway. Among genes assigned to at least one pathway, 93.5% are within 100 kb of a SNP. And 45% of SNPs map to at least one pathway gene (Figure S6, Panel D).

Computation for statistical analysis

The main difficulty in computing the Bayes factor (equations 3 and 5) is the combinatorially large number of ways we can include SNPs in the multi-marker disease model (eq. 1). In previous work [75], we described an approximation that yields an efficient procedure for computing marginal likelihoods and posterior quantities for this model. (This approximation was derived for the case when all variables have the same prior inclusion probability, or when $\theta = 0$, but it is straightforward to extend this approximation to the more general case, with $\theta \neq 0$.) Once we have a recipe for efficiently computing the likelihood $p(y \mid \mathbf{X}, a, \theta_0, \theta)$, we are left with the task of computing a one-dimensional integral in the denominator of the BF, and a double integral in the numerator. Each of these integrals is further approximated using simple numerical integration techniques.

The basic idea behind the approximation is to formulate a lower bound to the marginal likelihood,

$$p(y \mid \mathbf{X}, a, \theta_0, \theta) \geq e^{F(\mathbf{D}, \theta_0, \theta, \psi)}, \tag{9}$$

then to adjust the free parameters, denoted by ψ, so that this bound is as tight as possible. (The exact form of $F(\mathbf{D}, \theta_0, \theta, \psi)$ is derived in [75], and is reproduced below for convenience.) As before, $\mathbf{D} = \{\mathbf{X}, y, a\}$ is shorthand for the GWAS data. The lower bound is formulated by introducing a probability distribution $q(\beta; \psi)$ that approximates the posterior of β given θ_0 and θ. Maximizing this lower bound corresponds to finding the approximating distribution that best matches the posterior; more precisely, it amounts to searching for the free parameters ψ that minimize the Kullback-Leibler divergence between $q(\beta; \psi)$ and the posterior of β given θ_0 and θ [188]. The trick to making this approach tractable lies in forcing $q(\beta; \psi)$ to observe a simple conditional independence property, as suggested by [139]: each regression coefficient β_j is independent of the other coefficients *a posteriori* given θ_0 and θ. In other words, we restrict this distribution to be of the form

$$q(\beta; \psi) = \prod_{j=1}^{p} q(\beta_j; \psi_j), \tag{10}$$

where ψ_j is the set of free parameters corresponding to the jth factor.

For most SNPs, this conditional independence assumption is appropriate—most SNPs are unlinked because they are on separate chromosomes, or they are weakly linked because of recombination. In this case, the fully-factorized approximation $q(\beta; \psi)$ will closely recover the correct posterior distribution of the regression coefficients β_j for these SNPs. But the conditional independence assumption is violated for SNPs in linkage disequilibrium. In that case, we do not expect to obtain accurate posterior statistics, and, as a result, the lower bound (9) can be a poor substitute to the true likelihood. However, we are interested in accurate computation of BFs, not individual likelihoods, so what matters is whether $e^{F(\mathbf{D}, \theta_0, \theta, \psi)}$ correctly captures the *shape* of the likelihood, or how the likelihood changes as a function of θ_0 and θ. For example, if the lower bound undershoots the likelihood by a constant factor across different settings of θ_0 and θ, this constant factor will cancel out in the BF and in the posterior probabilities, leading to accurate estimates of these quantities. In [75], we show that the variational approximation—for the case when the phenotype is a quantitative trait—can closely reproduce the shape of the likelihood, and can give accurate estimates of some posterior quantities, even when the conditional independence assumptions are not appropriate. We caution, however, that the accuracy the approximate calculations has only been assessed empirically, and we do not have theoretical guarantees of their accuracy.

Computing the Bayes factor for one pathway

To compute the Bayes factor for a given pathway annotation a, we formulate a simple piecewise numerical approximation [189] to the 1-d and 2-d integrals in the numerator and denominator, and we replace each instance of the likelihood with its corresponding lower bound (9):

$$\begin{aligned} \mathrm{BF}(a) &= \frac{\iint p(y \mid \mathbf{X}, a, \theta_0, \theta)\, p(\theta_0)\, p(\theta)\, d\theta\, d\theta_0}{\int p(y \mid \mathbf{X}, a, \theta_0, \theta = 0)\, p(\theta_0)\, d\theta_0} \\ &\approx \frac{\iint e^{F(\mathbf{D}, \theta_0, \theta, \psi(\theta_0, \theta))}\, p(\theta_0)\, p(\theta)\, d\theta\, d\theta_0}{\int e^{F(\mathbf{D}, \theta_0, \theta = 0, \psi(\theta_0, \theta = 0))}\, p(\theta_0)\, d\theta_0}. \end{aligned} \tag{11}$$

Since the priors are uniform over θ_0 and θ, the numerical approximation to the Bayes factor works out to be

$$\mathrm{BF}(a) \approx \frac{I_{\mathrm{alt}}}{I_{\mathrm{null}}} = \frac{\frac{1}{n_1} \sum_{s=1}^{n_1} \exp\{F(\mathbf{D}, \theta_0^{(s)}, \theta^{(s)}, \psi(\theta_0^{(s)}, \theta^{(s)}))\}}{\frac{1}{n_0} \sum_{s=1}^{n_0} \exp\{F(\mathbf{D}, \theta_0^{(s)}, \theta = 0, \psi(\theta_0^{(s)}, \theta = 0))\}}. \tag{12}$$

To compute I_{null}, the numerical approximation to the null likelihood, we evaluate the lower bound at n_0 equally spaced points over interval $[-6, -2]$. To compute I_{alt}, the likelihood under the alternative, we evaluate the lower bound at n_1 points on a regular grid over rectangular region $\theta_0 \in [-6, -2]$, $\theta \in [0, 5]$.

Observe that the free parameters ψ in (12) are expressed as a function of θ_0 and θ. This is because we adjust ψ separately for each setting (θ_0, θ). Optimizing ψ involves iterating coordinate ascent steps until these steps converge to stationary point. This stationary point constitutes a locally optimal bound to the likelihood. Full details about the procedure to solve for $\psi(\theta_0, \theta)$ are given in [75]. This procedure scales linearly with the number of samples and the number of SNPs.

Adjusting the free parameters for each setting (θ_0, θ) can be an costly endeavor for a large data set, so to reduce the expense of computing BF(a) we formulate a piecewise numerical approximation to each integral using as few grid points as possible (the number of grid points is given below). A coarse partitioning can risk some loss of accuracy, especially if the posterior distribution of the hyperparameters is sharply peaked inside the subintervals. An adaptive method that refines the subintervals in the piecewise approximation could have been used instead [189], but we stick to this simple scheme with equally spaced points at larger subintervals because it allows us to obtain reasonably accurate estimates of BFs for all candidate pathways at a manageable computational cost.

The analytic expression for the lower bound to the log-likelihood is derived in the Appendix of [75] for $\theta = 0$.

For convenience, we reproduce the expression for the more general case, with $\theta \neq 0$:

$$
\begin{aligned}
F(\mathbf{D}, \theta_0, \theta, \psi) &= \log \hat{\sigma}_0 + \frac{\bar{y}^2}{2\bar{u}} + \sum_{i=1}^{n} \log \phi(\eta_i) + \tfrac{\eta_i}{2}(u_i \eta_i - 1) + \hat{y}^T \mathbf{X} r - \tfrac{1}{2} r^T \mathbf{X}^T \hat{U} \mathbf{X} r \\
&\quad - \frac{1}{2} \sum_{j=1}^{p} (\mathbf{X}^T \hat{U} \mathbf{X})_{jj} \mathrm{Var}[\beta_j] + \sum_{j=1}^{p} \frac{\alpha_j}{2} \left[1 + \log \left(\frac{s_j^2}{\sigma_a^2} \right) - \frac{s_j^2 + \mu_j^2}{\sigma_a^2} \right] \\
&\quad - \sum_{j=1}^{p} \alpha_j \log \left(\frac{\alpha_j}{\pi_j} \right) - \sum_{j=1}^{p} (1 - \alpha_j) \log \left(\frac{1 - \alpha_j}{1 - \pi_j} \right),
\end{aligned}
\tag{13}
$$

For this expression, we define the following notation: π_j is the prior inclusion probability for SNP j (eq. 2); α_j is the PIP for SNP j with respect to the approximating distribution $q(\beta; \psi)$; μ_j and s_j^2 are the approximate mean and variance of coefficient β_j conditioned on SNP j being included in the model; $\mathrm{Var}[\beta_j] = \alpha_j(\mu_j^2 + s_j^2) - (\alpha_j \mu_j)^2$ is the variance of β_j with respect to the approximating distribution; r is the column vector with entries $r_j = \alpha_j \mu_j$; $\hat{\sigma}_0 = 1/\sqrt{\bar{u}}$ is the standard deviation of the intercept β_0 given β; $\hat{\beta}_0 = \bar{y}/\bar{u}$ is the posterior mode of the intercept β_0 assuming $\beta = 0$; $(\mathbf{X}^T \hat{U} \mathbf{X})_{jj}$ is the jth diagonal entry of matrix product $\mathbf{X}^T \hat{U} \mathbf{X}$; $\bar{u} = \sum_{i=1}^n u_i$, $\bar{y} = \sum_{i=1}^n (y_i - \frac{1}{2})$, $\hat{y} = y - \frac{1}{2} - \hat{\beta}_0 u$, $\hat{U} = U - uu^T/\bar{u}$; u is a column vector with entries $u_i = (\phi(\eta_i) - \frac{1}{2})/\eta_i$; U is the $n \times n$ matrix with diagonal entries u_i; and $\phi(x)$ is the sigmoid function, defined in the main text. When we derived this analytic expression for the lower bound in [75], we made an additional approximation to the nonlinear factors appearing in the logistic regression likelihood $p(y \mid \mathbf{X}, \beta_0, \beta)$, following [190,191]. This approximation introduces an additional set of free parameters, $\eta = (\eta_1, \ldots, \eta_n)$, and the lower bound (13) is therefore a function of η as well. Like ψ, we adjust η separately for each (θ_0, θ). The procedure to solve for η is given in [75].

Since the coordinate ascent updates used to solve for ψ and η are only guaranteed to converge to a local maximum of the lower bound, the choice of starting point can affect the tightness of the lower bound, and the quality of the approximation. As we explain in [75], this issue can be addressed to some degree by using a common initialization $(\psi^{(\mathrm{init})}, \eta^{(\mathrm{init})})$ for the coordinate ascent updates across all grid points $(\theta_0^{(s)}, \theta^{(s)})$, in which this initialization is selected by first running the coordinate ascent procedure separately for each grid point, with random initializations for ψ and η, then assigning $(\psi^{(\mathrm{init})}, \eta^{(\mathrm{init})})$ to the solution from the hyperparameter setting with the largest lower bound. When we follow this procedure in practice, we find that the final estimates of the BFs and posterior statistics vary only slightly when the analysis is re-run with different random initializations. However, we cannot guarantee that a new random starting point will not produce a better approximation, and in practice, our numbers may not be reproduced exactly in an independent analysis using the same method, though we expect only slight deviations from our numbers.

Computing the posterior inclusion probabilities and other posterior statistics

In this section, we outline computation of posterior inclusion probabilities (PIPs) and other relevant posterior statistics when a pathway, or combination of pathways, is enriched. Computation of these quantities under the null hypothesis proceeds by setting $\theta = 0$.

Similar to the procedure for computing the BFs, we formulate a piecewise numerical approximation to the integral in (7), substituting each PIP conditioned on hyperparameter setting (θ_0, θ) with its corresponding variational approximation, $\alpha_j(\theta_0, \theta) \approx p(\beta_j \neq 0 \mid \mathbf{D}, \theta_0, \theta)$. This yields the approximate PIP

$$
\begin{aligned}
\mathrm{PIP}(j) &= \iint p(\beta_j \neq 0 \mid \mathbf{D}, \theta_0, \theta)\, p(\theta_0, \theta \mid \mathbf{D})\, d\theta_0\, d\theta \\
&\approx \textstyle\sum_{s=1}^{n_1} \alpha_j(\theta_0^{(s)}, \theta^{(s)})\, \tilde{w}_s,
\end{aligned}
\tag{14}
$$

where we define

$$
\tilde{w}_s \propto e^{F(\mathbf{D}, \theta_0^{(s)}, \theta^{(s)}, \psi(\theta_0^{(s)}, \theta^{(s)}))},
$$

such that $\sum_{s=1}^{n_1} \tilde{w}_s = 1$. Other posterior quantities are computed by averaging over θ_0 and θ in a similar way. For example, the posterior mean of the log-fold enrichment is

$$\begin{aligned} E[\theta \,|\, \mathbf{D}] &= \iint \theta \, p(\theta_0, \theta \,|\, \mathbf{D}) \, d\theta_0 \, d\theta \\ &\approx \textstyle\sum_{s=1}^{n_1} \theta^{(s)} \, \tilde{w}_s. \end{aligned} \tag{15}$$

To compute 95% credible intervals for θ, we add up the normalized weights $\tilde{w}_s$ over successively wider intervals of θ, beginning at the posterior mean, until the sum of the normalized weights $\tilde{w}_s$ reaches 0.95. Note that the credible interval is at the same resolution as the grid points used for the numerical approximation. The credible interval is not necessarily symmetric about the mean; other definitions of the credible interval are possible [192].

The numerical estimate of P_1, the posterior probability that at least 1 SNP in a given region of the genome is included in the model, is

$$\begin{aligned} P_1 &= \iint p(S \geq 1 \,|\, \mathbf{D}, \theta_0, \theta) \, p(\theta_0, \theta \,|\, \mathbf{D}) \, d\theta_0 \, d\theta \\ &\approx \textstyle\sum_{s=1}^{n_1} p(S \geq 1 \,|\, \mathbf{D}, \theta_0^{(s)}, \theta^{(s)}) \, \tilde{w}_s \\ &= \textstyle\sum_{s=1}^{n_s} \left[1 - p(S = 0 \,|\, \mathbf{D}, \theta_0^{(s)}, \theta^{(s)})\right] \tilde{w}_s, \end{aligned} \tag{16}$$

where $S = n$ denotes the event that exactly n SNPs in the region are included. Let the SNPs in the segment be labeled 1 through m. Since the regression coefficients are independent under the approximating distribution, for any (θ_0, θ) we have that

$$\begin{aligned} p(S = 0 \,|\, \mathbf{D}, \theta_0, \theta) &= p(\beta_1 = 0 \wedge \cdots \wedge \beta_m = 0 \,|\, \mathbf{D}, \theta_0, \theta) \\ &\approx \textstyle\prod_{j=1}^{m} q(\beta_j = 0; \psi(\theta_0, \theta)) \\ &= \textstyle\prod_{j=1}^{m} (1 - \alpha_j(\theta_0, \theta)). \end{aligned} \tag{17}$$

Therefore, the final estimate of the posterior statistic P_1 averaged over settings of the hyperparameters is

$$P_1 \approx \textstyle\sum_{s=1}^{n_s} \tilde{w}_s \left[1 - \prod_{j=1}^{m} (1 - \alpha_j(\theta_0^{(s)}, \theta^{(s)}))\right]. \tag{18}$$

To compute $P_2 = p(S \geq 2 \,|\, \mathbf{D})$ for a given segment, we observe that $p(S \geq 2) = 1 - p(S = 1) - p(S = 0)$. Thus, under the fully-factorized variational approximation, we have that

$$\begin{aligned} p(S = 1 \,|\, \mathbf{D}, \theta_0, \theta) &= p(\beta_1 \neq 0 \wedge \beta_2 = 0 \wedge \cdots \wedge \beta_m = 0 \,|\, \mathbf{D}, \theta_0, \theta) \\ &\quad + p(\beta_1 = 0 \wedge \beta_2 \neq 0 \wedge \cdots \wedge \beta_m = 0 \,|\, \mathbf{D}, \theta_0, \theta) \\ &\quad + \cdots \\ &\quad + p(\beta_1 = 0 \wedge \cdots \wedge \beta_{m-1} = 0 \wedge \beta_m \neq 0 \,|\, \mathbf{D}, \theta_0, \theta) \\ &\approx \left[\prod_{j=1}^{m} (1 - \alpha_j)\right] \times \left[\sum_{j=1}^{m} \frac{\alpha_j}{1 - \alpha_j}\right], \end{aligned} \tag{19}$$

where α_j in this expression is shorthand for $\alpha_j(\theta_0, \theta)$.

Scaling computation to many pathways, and modifications to computation to account for large contributions of MHC alleles to RA and T1D risk

Numerical integration together with the variational approximation now makes it feasible to compute a Bayes factor for one pathway. However, computing BFs for thousands of candidate pathways is still a costly and time-consuming undertaking. Next we introduce a simplifying assumption that allows us to reuse our computations, yielding a substantial reduction in the computational complexity of computing BFs for all candidate pathways.

We make the assumption that SNPs outside the enriched pathway are unaffected by pathway enrichment *a posteriori*. Formally, we assume

$$p(\beta_{\bar{A}} \,|\, \mathbf{D}, \theta_0, \theta) = p(\beta_{\bar{A}} \,|\, \mathbf{D}, \theta_0, \theta = 0), \tag{20}$$

where A is the set of SNPs assigned to the enriched pathway, and $\bar{A}$ is the remaining set of SNPs. In other words, the posterior distribution of the regression coefficients for SNPs outside the enriched pathway—which is usually most SNPs—remains the same under the null and enrichment models. With this assumption, the posterior distribution of β given θ_0, θ factorizes as

$$p(\beta \,|\, \mathbf{D}, \theta_0, \theta) \approx p(\beta_A \,|\, \mathbf{D}, \theta_0, \theta, \beta_{\bar{A}})\, p(\beta_{\bar{A}} \,|\, \mathbf{D}, \theta_0, \theta = 0). \tag{21}$$

This assumption amounts to conditioning on the effects of SNPs outside the enriched pathway estimated under the null model.

It is of course possible that SNPs contributing evidence for pathway enrichment are correlated with SNPs outside the pathway, invalidating this assumption. But because we assign SNPs to pathways in contiguous blocks (see Methods), and because the way we assign SNPs to genes is not precise (many SNPs assigned to a gene are probably not relevant to the gene), errors as a result of this assumption are expected to be minor relative to imprecision of the SNP-pathway assignments. If we were to assign SNPs to pathways more precisely, then this assumption would be less appropriate.

Next, we show how this assumption, combined with the variational approximation, allows us to reuse computations. Suppose we have two settings of the hyperparameters, (θ_0, θ) and (θ_0^*, θ^*). Further suppose that for any SNP outside the enriched pathway, the approximate marginal posterior $q(\beta_j; \psi_j)$ remains the same under both hyperparameter settings; that is, for any $j \notin A$, $\psi_j = \psi_j^*$, in which $q(\beta; \psi)$ approximates the posterior distribution of β given (θ_0, θ), and $q(\beta; \psi^*)$ approximates the posterior distribution of β given (θ_0^*, θ^*). Given these assumptions, it can be shown that the variational lower bound satisfies

$$F(\mathbf{D}, \theta_0, \theta, \psi) - F_A(\{\mathbf{X}, \hat{y}_A, a\}, \theta_0, \theta, \psi) = F(\mathbf{D}, \theta_0^*, \theta^*, \psi^*) - F_A(\{\mathbf{X}, \hat{y}_A, a\}, \theta_0^*, \theta^*, \psi^*), \tag{22}$$

in which we introduce the following definitions: $F_A(\mathbf{D}, \theta_0, \theta, \psi)$ is the expression for the lower bound that only accounts for SNPs in A and ignores all other SNPs; $\hat{y}_A = y - \hat{U}\mathbf{X}_{\bar{A}} r_{\bar{A}}$ is the vector of binary labels "corrected" for the effects of SNPs outside the pathway; $\mathbf{X}_{\bar{A}}$ is the matrix of genotypes that contains one column for each SNP $j \notin A$, and $r_{\bar{A}}$ is the vector r with entries $r_j = \alpha_j \mu_j$ restricted to $j \notin A$. Note (22) is valid only if η is held constant.

Identity (22) suggests a way to reuse our computations: once we have solved for $\psi(\theta_0, \theta = 0)$, the free parameters ψ that (locally) maximize the lower bound under the null hypothesis for a given θ_0, to solve for $\psi(\theta_0, \theta)$ for any $\theta \neq 0$, we only need to adjust the free parameters ψ_j for SNPs j assigned to the enriched pathway. Crucially, η must be held constant in (22), so for any $\theta \neq 0$, we set $\eta(\theta_0, \theta) = \eta(\theta_0, \theta = 0)$.

Following similar logic, the variational approximation is easily adapted to the modified analysis for RA and T1D (described in Methods). In the first step, we fit the multi-marker model using only SNPs outside the MHC. In the second step, we fit the disease model using all available SNPs, in which the regression coefficients of non-MHC SNPs remain fixed. When conditioning on the effects of non-MHC SNPs in the second step, we account for uncertainty in their estimates by averaging over possible assignments to the regression coefficients. This is accomplished by adjusting the free parameters ψ_j only for SNPs j inside the MHC, and replacing the vector of observations y by $\hat{y} = y - \hat{U}\mathbf{X}_B r_B$, where B is the set of SNPs outside the MHC.

More details on analysis of seven common diseases

Here we provide additional details about analysis of the data sets for bipolar disorder (BD), coronary artery disease (CAD), Crohn's disease (CD), hypertension (HT), rheumatoid arthritis (RA), type 1 diabetes (T1D), and type 2 diabetes (T2D). For complete details about our analysis procedures, consult the MATLAB source code, which we have made available at `http://github.com/pcarbo/bmapathway`. Table S1 summarizes the genome-wide marker data from the seven case-control studies [65].

Multi-marker disease mapping under null model

The first step in the analysis is to fit the multi-marker disease model to the data, ignoring any information about pathways. This step is important for two reasons: one, to determine which regions of the genome are relevant to disease solely based on a multi-marker analysis of the data, so that we can later appreciate what is gained by incorporating pathways into the analysis; two, to calculate posterior quantities that will be reused to efficiently compute BFs for all candidate pathways.

An important advantage of joint analysis of SNPs using a multi-marker model is that the priors are automatically guided by the data. In the absence of enriched pathways, the prior inclusion probability for all SNPs is governed by a single parameter, θ_0 (eq. 2). Rather than compute a single estimate for this parameter, we account for possible uncertainty in θ_0 by evaluating the posterior probability of θ_0 at equally spaced points between -6 and -2. We begin with a coarse grid of points: we evaluate the posterior probability $p(\theta_0 \,|\, \mathbf{X}, y)$—or, more precisely, an approximation to this posterior probability obtained by tightening the variational lower bound to the marginal likelihood—at equally spaced points $\theta_0 = -6, -5.75, \ldots, -2$. Once we identify a range where most of the posterior mass for θ_0 appears to lie, we redo the analysis using a finer grid, at intervals of 0.1. In this way, we obtain accurate posterior estimates of θ_0, while reducing the number of times we repeat the computationally intensive step of computing the variational approximation to the marginal likelihood. For example, in the analysis of the CD data, we initially find that most of the posterior mass for θ_0 lies between -4.5 and 3, so we can reasonably ignore settings of θ_0 outside that range, and focus on computing posterior probabilities $p(\theta_0 \,|\, \mathbf{X}, y)$ at points $\theta_0 = -4.5, -4.4, \ldots, -3$. These posterior probabilities are later used to formulate piecewise numerical approximations to the integrals over θ_0, for example to compute PIPs.

Once we complete this step, we record the posterior mean and 95% credible interval of θ_0 for each disease (Figure S7). The wide credible intervals for BD, CAD, HT and T2D reflect a weak association signal across the genome, hence little information about θ_0. In the other diseases, we have more information about θ_0 thanks to stronger genome-wide association signals. Parameter θ_0 gives us a rough estimate of the expected number of SNPs that are independently associated with disease status. For instance, given posterior mean estimate $\theta_0 = -3.7$ for CD, roughly $442,001 \times 10^{-3.7} = 88$ SNPs are expected to be included in the multi-marker disease model *a priori.* In practice, of course, we identify a much smaller number of regions relevant to disease because only a few SNPs are included in the model with high probability.

Posterior estimates for θ_0 in RA and T1D are obtained using the modified analysis, in which we initially fit the multi-marker model using SNPs outside the MHC, as explained in Methods. In the modified analysis, the posterior mean of θ_0 is -4.3 for RA, and -4.8 for T1D (Figure S7), whereas the posterior means are -4.0 and -3.8 when we fit all SNPs simultaneously. It is possible that discrepancies in these posterior estimates occur because the modified analysis, in effect, does not let the co-ordinate ascent updates for optimizing the variational approximation run to completion, so we obtain a suboptimal variational approximation that does not capture the likelihood surface of θ_0 as accurately as the unmodified analysis. (Note that we have not yet incorporated enrichment of the MHC into our analysis at this stage.)

Now that we have calculated the posterior distribution of θ_0, we proceed to obtain posterior statistics for individual SNPs by numerically integrating over θ_0. To locate regions of the genome relevant to disease it is preferable to compute P_1 over blocks of SNPs (see Results). Specifically, we divide the genome into segments of 50 SNPs, with an overlap of 25 SNPs between neighbouring segments. The genome-wide scans derived by computing P_1 for each 50-SNP segment are shown in Figures S8 and S9. We observe that the association signal from multi-marker mapping is more sparse than a typical genome-wide scan, in which SNPs are tested one at a time for association with the disease. In a single-SNP regression analysis, SNPs in a relevant region that are correlated with one another will all show strong evidence for association, whereas the multi-marker analysis accounts for correlations between markers, so nearby markers that are strongly correlated with each other do not simultaneously exhibit a strong association signal. For example, within a block of highly correlated SNPs, usually at most one SNP in the block will be included in the multi-marker model.

The 50-SNP segments with $P_1 \geq 0.5$ are listed in Table S2. The selected segments overlap with the regions highlighted in the original study [65]. Note that the actual disease-conferring variant(s) may not lie within the

50-SNP segment with $P_1 \geq 0.5$, depending on the extent of linkage disequilibrium among markers in the region. (For segments overlapping the MHC, the disease risk variants may lie far away from the segment with high P_1 due to long-range correlations between markers in the MHC.) In practice, it is preferable to report the region that most likely contains the risk-conferring variant(s), and this can be estimated by inspecting the association signal and pattern of correlations among markers in the region, but in Table S2 we show instead the region spanned by the 50-SNP segment for the purpose of describing the steps in our analysis.

In three diseases, CD, RA and T1D, we obtain moderate to strong support for disease variants mapping to the MHC. A large number of MHC SNPs are included in models for RA and T1D (the expected number of included SNPs is 7.1 for RA, and 13.6 for T1D). Although the individual effects of SNPs are not large, the combined effect is, so genetic contributions to disease susceptibility within the MHC may hamper our ability to accurately estimate smaller, non-MHC effects when jointly analyzing SNPs, following [162], and as we explain in Methods. In contrast, for CD the expected number of included SNPs within the MHC is low (0.91), and the estimated effects of these included SNPs are no larger than the effects of SNPs elsewhere in the genome. Therefore, we expect that MHC alleles do not substantially affect power to detect non-MHC associations with CD.

To relate the disease association results using a multi-marker model to the original results based on single-marker regression, we show two scatterplots comparing our posterior statistics to the trend p-values and Bayes factors given in [65]. Overall, the multi-marker results correspond well to the original association results (Figure S4): the largest trend p-value corresponding to the selected disease regions (Table S2) is 8.65×10^{-7}, and the smallest corresponding additive BF is 4.19; the selected regions capture all associations with trend p-values less than 5.1×10^{-8}, or additive BFs greater than $10^{5.36}$ in [65]. Among points in the scatterplots that depart appreciably from the diagonal (Figure S4), most show greater support for association in the multi-marker analysis. Only one disease association, locus 5q33 in the analysis of CD, shows noticeably stronger support in the single-marker analysis; our calculations yield a largest P_1 of 0.21 at this locus. (Note that SNPs in this region is not assigned to any of the pathways that show strong evidence for enrichment of CD associations.) This CD association is replicated elsewhere [6, 7, 87], and has implicated *IRGM*, an autophagy gene, in CD pathogenesis [5]. Finally, we point out that the SNP with the largest PIP in a given genome segment is not necessarily the same as the SNP with the largest p-value or Bayes factor in the single-marker analysis (Table S2).

The association results for RA are largely unchanged in the modified analysis, whereas we obtain different association results for T1D in the modified and unmodified analyses (Figure S5). The T1D association results from the modified analysis better coincide with the original single-marker association results (Figure S4). As expected, the results for other diseases remain largely unchanged if we analyze MHC and non-MHC SNPs separately (results not shown).

Bayes factors for enriched gene sets

Next we quantify support for pathway enrichment in all seven diseases. The first step is to narrow the search for enriched gene sets by calculating rough estimates of BFs for all candidate gene sets. For this step, we formulate numerical approximations to the integrals at equally spaced grid points $\theta_0 = -6, -5.5, \ldots, -2$ and $\theta = 0, 0.5, \ldots, 3$. Figure S2 summarizes the BFs for all candidate gene sets in all diseases.

Once we identify the most promising candidate pathways with the largest BFs, we refine the numerical estimates of the top BFs using a more finely spaced grid, with θ_0 evaluated over its full range, $[0, 5]$. For example, for CD we find that 19 pathways have BF greater than 100 based on our initial calculations. We compute more refined numerical estimates of the BFs for these pathways using equally spaced grid points, $\theta_0 = -5, -4.9, \ldots, -3$ and $\theta = 0, 0.1, \ldots, 5$. For the other diseases, we recompute BFs using the same grid for the following pathways: 4 pathways with BF > 10 in BD; 7 pathways with BF > 50 in CAD; 20 pathways with BF > 2 in HT; 5 pathways with BF > 100 in T2D; and for RA and T1D, we obtain by far the most support for enrichment of the MHC and xMHC, so we recompute more accurate BFs for the MHC and xMHC, as well as a few other pathways with the largest BFs. The initial numerical estimates of the BFs closely match the BFs calculated using the more finely spaced grid (Figure S10).

For RA and T1D, once we establish that the MHC has by far the most support for enrichment of disease

associations, we repeat the same steps just described, except that the null and alternative models now include enrichment of the MHC. Therefore, the BFs are now defined with respect to the null that the MHC is enriched. The two histograms in the bottom-right corner of Figure S2 summarize the BFs for the 3158 candidate gene sets conditioned on enrichment of the MHC. Once we obtain initial numerical estimates of these BFs, we compute more accurate estimates for the 4 pathways with BF > 100 in RA, and for the 17 pathways with BF $> 2 \times 10^8$ in T1D. These BFs are calculated with respect to the null hypothesis that the MHC is enriched for disease associations, but in Figure S1 we multiply these BFs by the BF for enrichment of the MHC to obtain a BF with respect to the null hypothesis with no enrichment. We also test the hypothesis that genes in the extended MHC that are outside the classical MHC are enriched at a different rate than classical MHC genes. Our calculations do not support this hypothesis; we obtain BFs of 0.54 and 0.64 for RA and T1D, respectively (to be clear, these BFs are defined relative the null hypothesis that the MHC is enriched). Because the MHC explains a considerable fraction of the association signal in RA and T1D, allowing for enrichment of the MHC effectively removes MHC associations from the "background", thereby producing lower estimates of the genome-wide rate of associations, θ_0; compare posterior mean estimates of θ_0 in Figure S7 against those in Figure 2). This, in turn, leads to larger estimates of θ in enriched pathways.

See Figure S1 for an expanded list of gene set enrichment results in diseases showing greatest support for pathway enrichment.

Bayes factors for combinations of enriched pathways

A benefit of our approach is that we can compare support for enrichment of different combinations of pathways by comparing their BFs (assuming the same prior for these enrichment hypotheses). This is because the ratio $\mathrm{BF}(a)/\mathrm{BF}(a^*)$ is the same as the BF that compares support for the enrichment model encoded by pathway annotation a versus the model encoded by a^*. (By contrast, it is harder to make such comparisons using p-values. For example, if p is the p-value for testing enrichment hypothesis a against the null, and p^* is the p-value for testing a^* against the null, it is not clear how to compare support for a and a^*.) We observe, for example, that the model in which cytokine signaling and IL-23 signaling genes are jointly enriched for CD associations is about 400 times greater than the BF for enrichment of cytokine signaling genes alone.

We follow the same procedures described in the previous section to compute BFs for enrichment models in which 2 or 3 pathways are enriched for disease associations.

Associations informed by enriched pathways

In this section, we point out some additional features of Figure 3.

The top enrichment result for T1D involves a relatively small set of IL-2 signaling genes (52 genes) that are highly upweighted; the prior inclusion probability for any SNP assigned to the pathway increases roughly 2000-fold relative to the null; the (expected) prior probability is 0.000016 under the null, and 0.030 under the alternative. By comparison, the increase in the prior is much smaller under enrichment of cytokine signaling (35-fold) and measles genes (188-fold).

Points in the top-right corner of each scatterplot correspond to regions with strong evidence for association even without the benefit of feedback from pathway enrichment. For example, genes *IL23R*, *PTGER4*, *ZNF365* and *NKX2-3* are not involved in cytokine signaling, nor are any nearby genes, so the SNPs near these genes are mostly unaffected by the hypothesis that the cytokine signaling pathway is enriched for CD associations. *NOD2* (also *CARD15*) and *PTPN2* are cytokine signaling genes, so these associations contribute to the evidence for enrichment of this pathway, but because they already show strong support for association without enrichment—that is, P_1 is close to 1 under the null—these associations are not noticeably affected by enrichment. An exception is *ATG16L1*, which is close enough to cytokine signaling gene *SHIP1* to be included in the same 50-SNP segment. However, the association signal in this case does not overlap with SNPs assigned to *SHIP1*, so it does not contribute to the evidence for enrichment of the cytokine signaling pathway.

Segments in the top-middle portion of each scatterplot originally show moderate evidence for association without pathways, and once we account for pathway enrichment, the evidence for associations becomes more compelling. Examples include CD associations in the MHC, and a T1D association near gene *CLEC16A*. These disease associations are well-replicated [7, 89, 130, 132, 183].

Two loci at 16q24 and 10q22 near pathway genes *IRF8* and *CAMK2G* harbour CD risk variants with probability just below our criterion for being included in Table 1 ($P_1 = 0.46$ for 16q24, and $P_1 = 0.43$ for 10q22). Both loci have been linked to CD in a recent meta-analysis of genome-wide IBD data sets [132].

Associations informed by multiple enriched pathways

We also investigate whether enrichment of multiple pathways can lead to identification of additional loci affecting susceptibility to disease. Conditioning on 2 enriched pathways in CD, RA and T1D does not yield strong support for genetic associations beyond those already revealed by enrichment of the single top pathway (Figure S3). (Since no single combination of pathways stands out in the rankings for pairs of pathways, P_1 conditioned on enrichment of 2 pathways is calculated by averaging over different enrichment models with the largest BFs; see Methods.) We do find, however, that a single 50-SNP segment shows an appreciable gain in support for association with CD, in which P_1 increases from 0.03 to 0.44. This segment spans 158.58–158.86 Mb on chromosome 5, as well as the coding sequence of *IL12B*. This region is reported as a CD association in other GWAS [6, 7, 132, 133]. When allowing for models in which 3 pathways are enriched, the probability that the region harbours CD risk-conferring variants increases to $P_1 = 0.57$.

Additional results from sensitivity analysis

Under the null hypothesis, there is a clear trend in the overall effect of the choice of σ_a on the distribution of associations in the CD data set—the posterior mean of θ_0 increases as σ_a decreases (Figure S11). For the vast majority of pathways, however, support for enrichment does not change noticeably with different settings of σ_a (Figure S12).

References

187. Cho M, Kang J, Moon Y, Nam H, Jhun J, et al. (2006) STAT3 and NF-kappaB signal pathway is required for IL-23-mediated IL-17 production in spontaneous arthritis animal model IL-1 receptor antagonist-deficient mice. Journal of Immunology 176: 5652–5661.

188. Jordan MI, Ghahramani Z, Jaakkola TS, Saul LK (1999) An introduction to variational methods for graphical models. Machine Learning 37: 183–233.

189. Burden R, Faires JD (2005) Numerical analysis. Thomson Brooks/Cole.

190. Bishop CM (2006) Pattern Recognition and Machine Learning. Springer.

191. Jaakkola TS, Jordan MI (2000) Bayesian parameter estimation via variational methods. Statistics and Computing 10: 25–37.

192. Hyndman RJ (2012) Computing and graphing highest density regions. American Statistician 50: 120–126.
